# Supplementary figures and images for: The actin module of endocytic internalization in Aspergillus nidulans: A critical role of the WISH/DIP/SPIN90 family protein Dip1
Source: PLoS Genet. 2025 Aug 26;21(8):e1011619. doi: 10.1371/journal.pgen.1011619 (PMC12422587; doi:10.1371/journal.pgen.1011619)

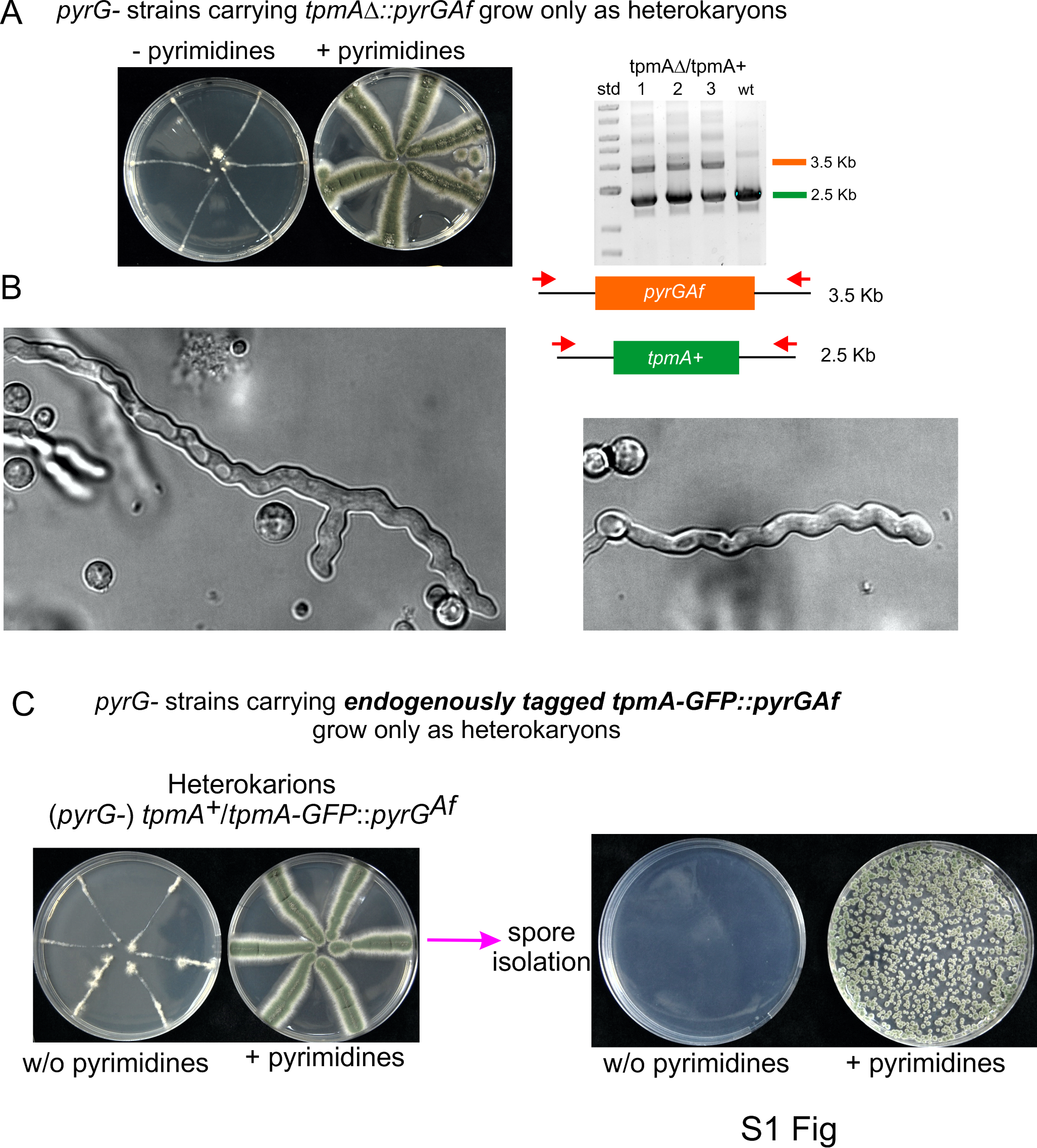

Supplement: S1 Fig — (A) Heterokaryon rescue of tpmAΔ. Heterokaryotic strains carrying pyrG89 tpmA+ and pyrG89 tpmAΔ::pyrGAf nuclei were generated by transformation. Uninucleate, haploid spores derived from these strains were able to grow when supplemented with pyrimidines, but not in their absence, as the ability to grow without them is linked to a lethal tpmAΔ::pyrGAf allele (pyrGAf. Is the A. fumigatus pyrG gene complementing pyrG89). Left, replicas of several of these strains in media supplemented or not with pyrimidines. Right, PCR genotyping of three such strains using tpmA flanking primers demonstrating the presence of both tpmA+ and tpmAΔ::pyrGAf in them. (B) tpmAΔ::pyrGAf conidiospores were able to established polarity but arrested growth shortly after, showing morphogenetically defective curly hyphae. (C) Heterokaryon rescue test as above showing that strains carrying tpmA endogenously tagged with GFP are unable to grow as homokaryons. (TIF) [file pgen.1011619.s001.tif]

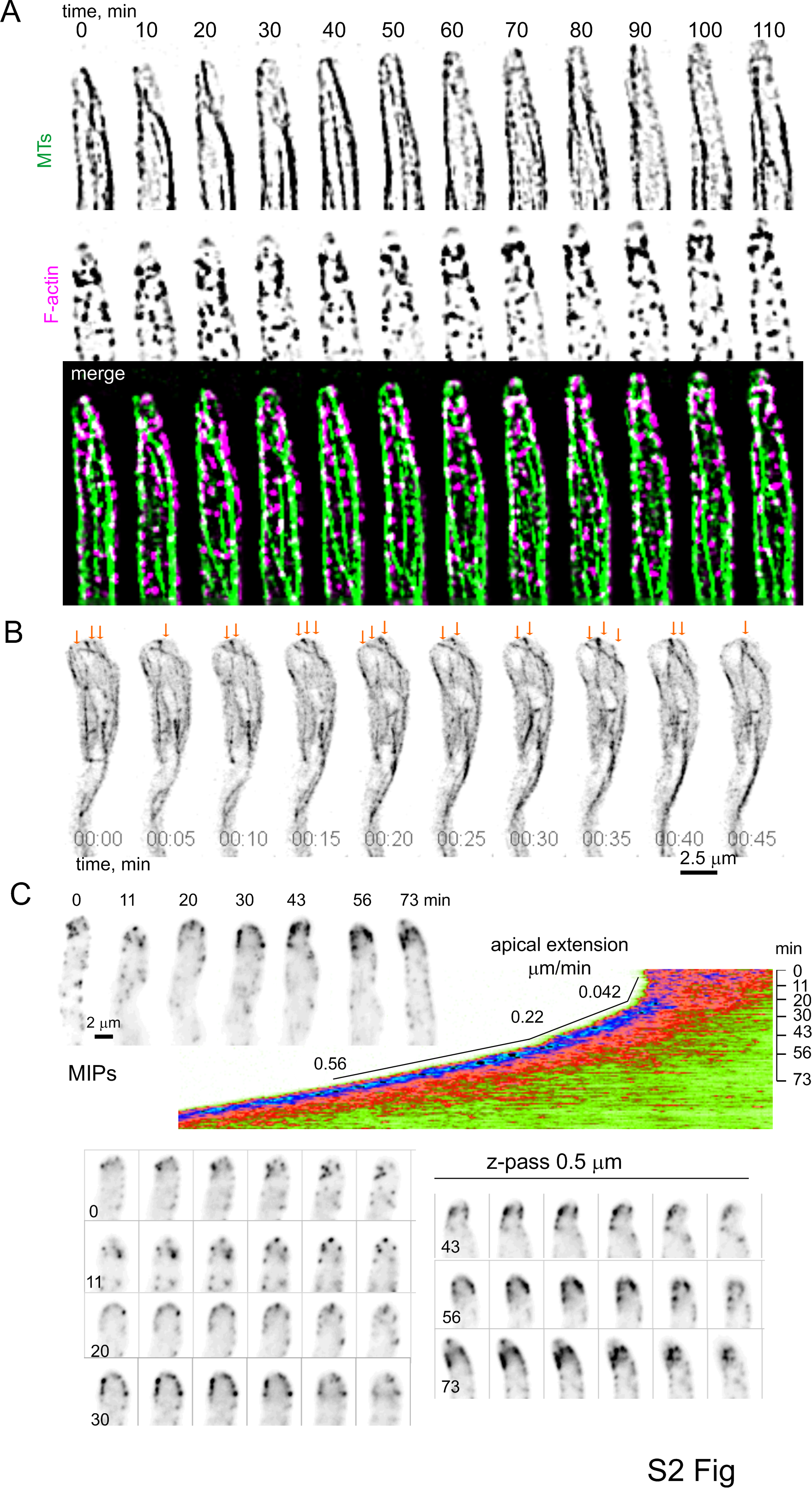

Supplement: S2 Fig — (A) Frames of a time-lapse sequence showing numerous instances in which MTs reach the SPK and its intermediate neighborhood. MTs are labeled with GFP-tagged alpha tubulin whereas actin was visualized with Lifeact-tdT. Images were deconvolved and further contrasted using the unsharp mask filter of Metamorph (B) A hyphal tip cell expressing GFP-tagged alpha-tubulin that had been incubated for 60 minutes with 50 μM latrunculin B. Contacts of the plus-ends of MTs with the cortex, indicated with orange arrows, are distributed all across the apical dome. (C) Recovery of the actin cytoskeleton after having been disorganized by depolymerization of MTs with benomyl (5 μM). The first frame of the movie corresponds to the six minute timepoint after washing out the drug. Top left, MIPs of Z-stacks back acquired at the indicated time points after washing out benomyl. Top, right, kymograph obtained with a longitudinal line ROI covering the whole width of the hypha, with deep blue representing the strongest signal and green the weakest. Changes in the rate of apical extension are noted. Bottom, individual sections of the Z-stacks used for MIPs are shown. These data have been extracted from S3 Movie. (TIF) [file pgen.1011619.s002.tif]

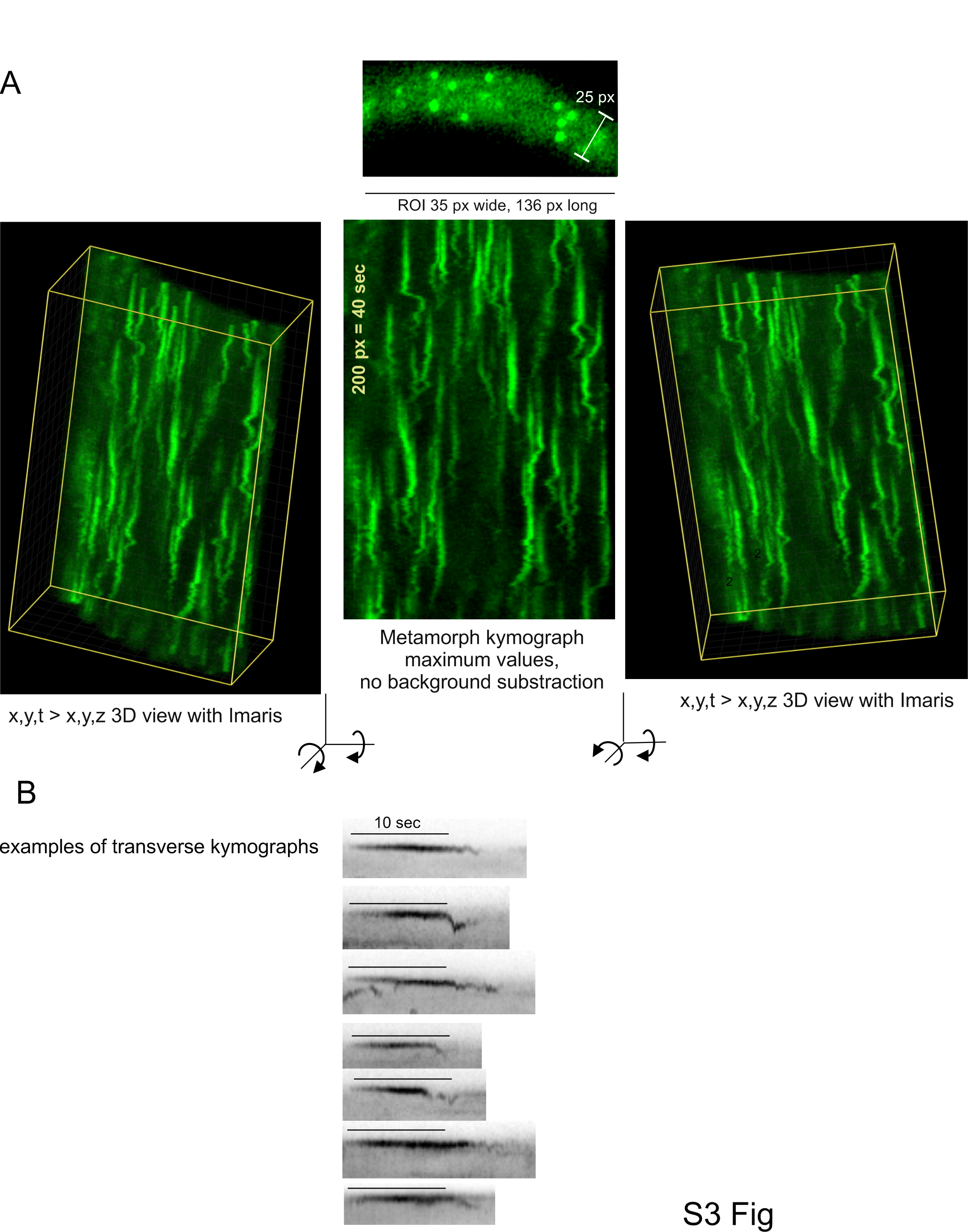

Supplement: S3 Fig — (A) Kymographs were usually obtained with linear ROIs traced longitudinally across the hyphae. ROIs were usually 12–25 pixel wide. If, as shown in this example, traces of individual endocytic events excessively overlapped, time stacks were manipulated as Z-stacks using Imaris software, which facilitated detection of individual traces. Details are also provided under Materials and Methods. (B) Examples of transverse kymographs. (TIF) [file pgen.1011619.s003.tif]

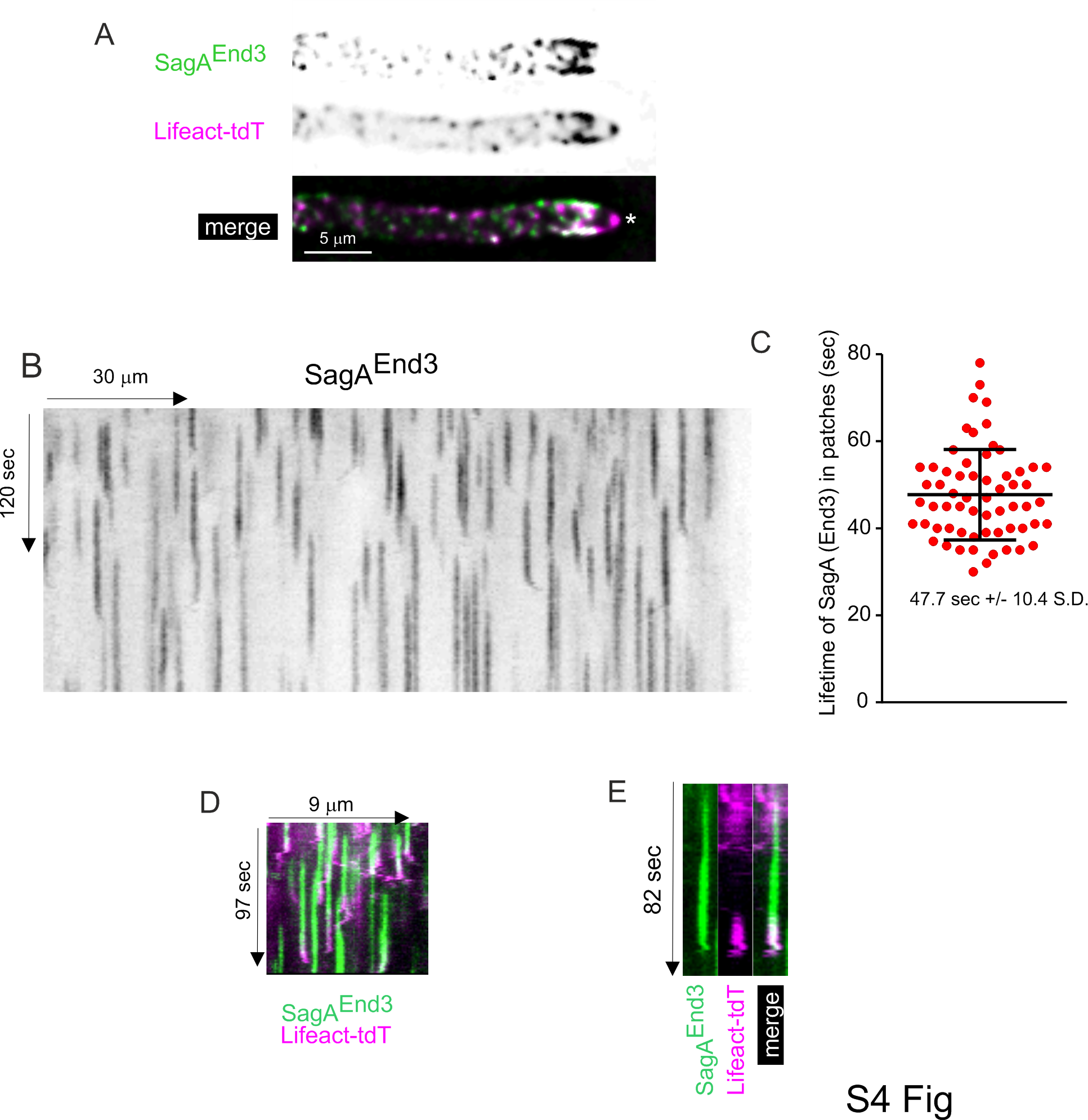

Supplement: S4 Fig — (A) middle planes of deconvolved Z-stacks showing that SagAEnd3 localizes to endocytic patches but not to the SPK. (B) and (C) Kymograph analysis of the time of residence of SagAEnd3 in endocytic patches. Error bars represent the means ± S.D. (D) Region of a kymograph obtained with a strain coexpressing GFP-tagged SagAEnd3 and Lifeact tdT. (E) Example of an actin patch from the above strain. (TIF) [file pgen.1011619.s004.tif]

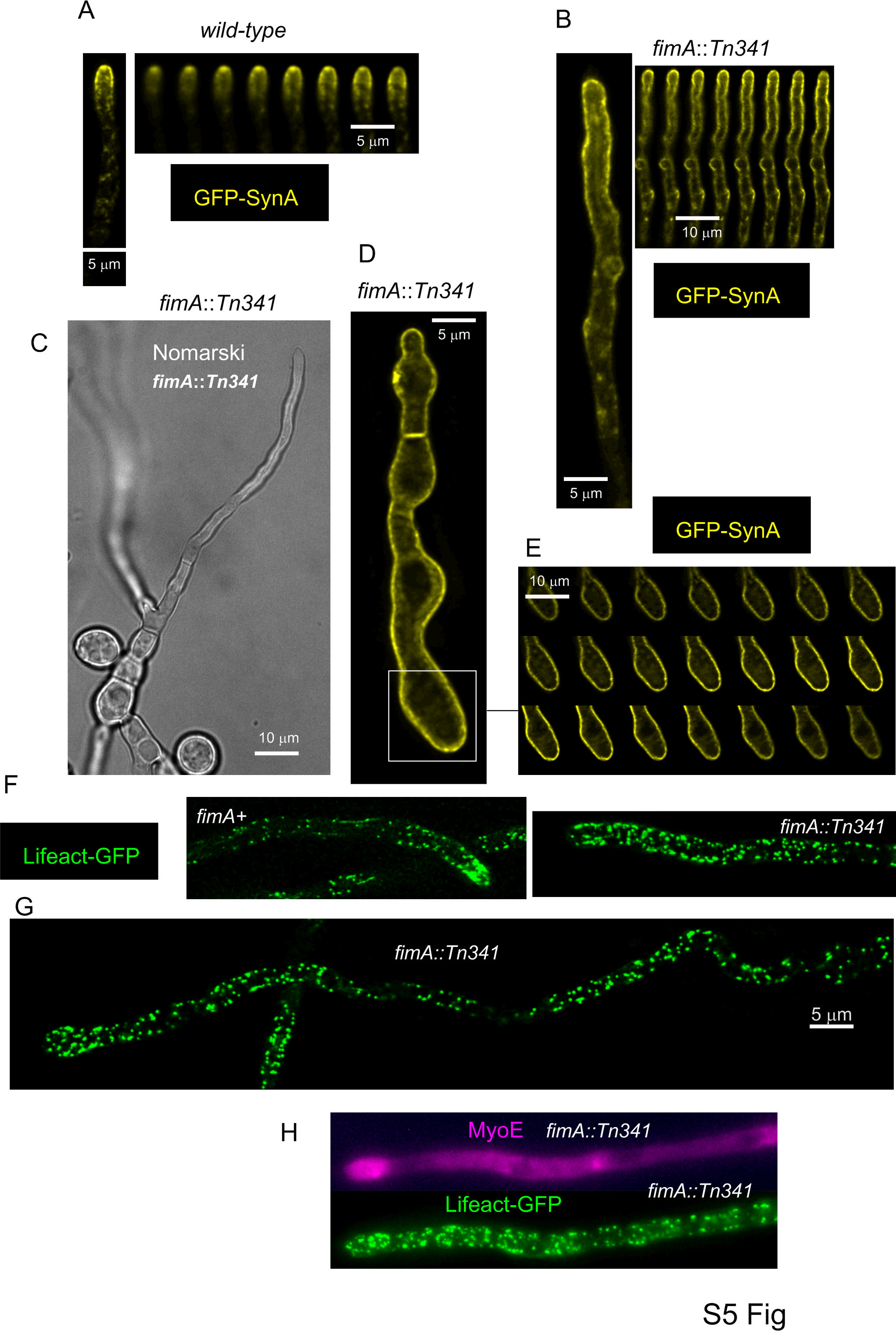

Supplement: S5 Fig — (A) MIP and corresponding Z-stack of a wild-type hyphal tip cell expressing GFP-SynA. (B) As in (A) for a fimA::Tn341 cell. (C) Morphological transitions following germination of a fimA::Tn341 conidiospore. (D) Uniform distribution of GFP-SynA in a markedly abnormal fimA::Tn341 germling. MIP. (E) Individual sections of the region boxed in (D) showing the absence of any remnant of SPK. (F) Lifeact-GFP distribution in a fimA::Tn341 cell which is morphogenetically normal compared to the wild type. (G) After a period of normal morphology fimA::Tn341 hyphae became swollen in the tips and arrested growth. (F) MyoE was completely delocalized to the cytosol in these fimA::Tn341 hyphae incubated for very long periods. (TIF) [file pgen.1011619.s005.tif]

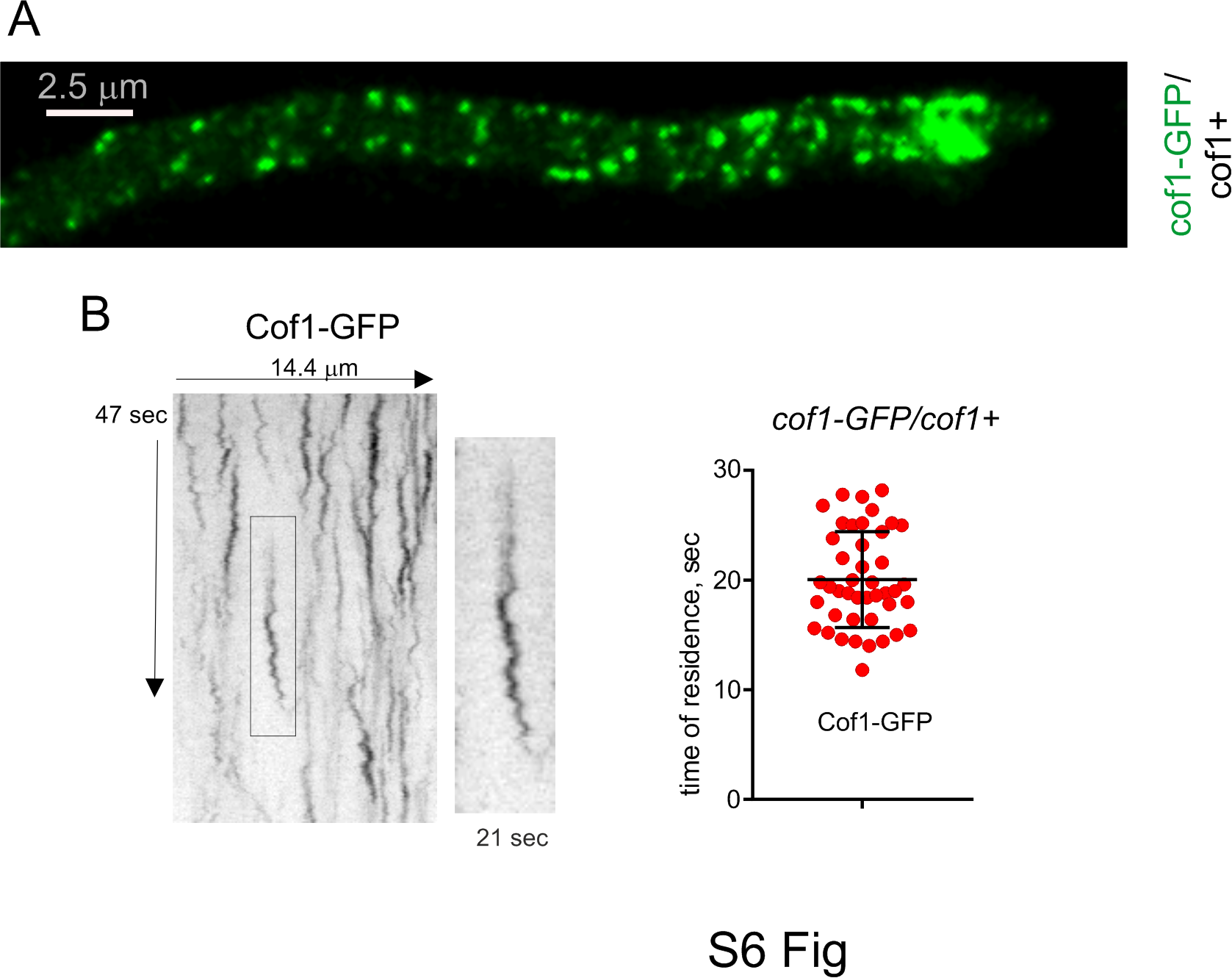

Supplement: S6 Fig — (A) MIP of a deconvolved Z-stack of Cof1-GFP expressed in a heterozygous diploid with the wild -type allele to maintain viability. (B) Analysis of Cof1-GFP endocytic patches. Error bars represent the mean ± S.D. (TIF) [file pgen.1011619.s006.tif]

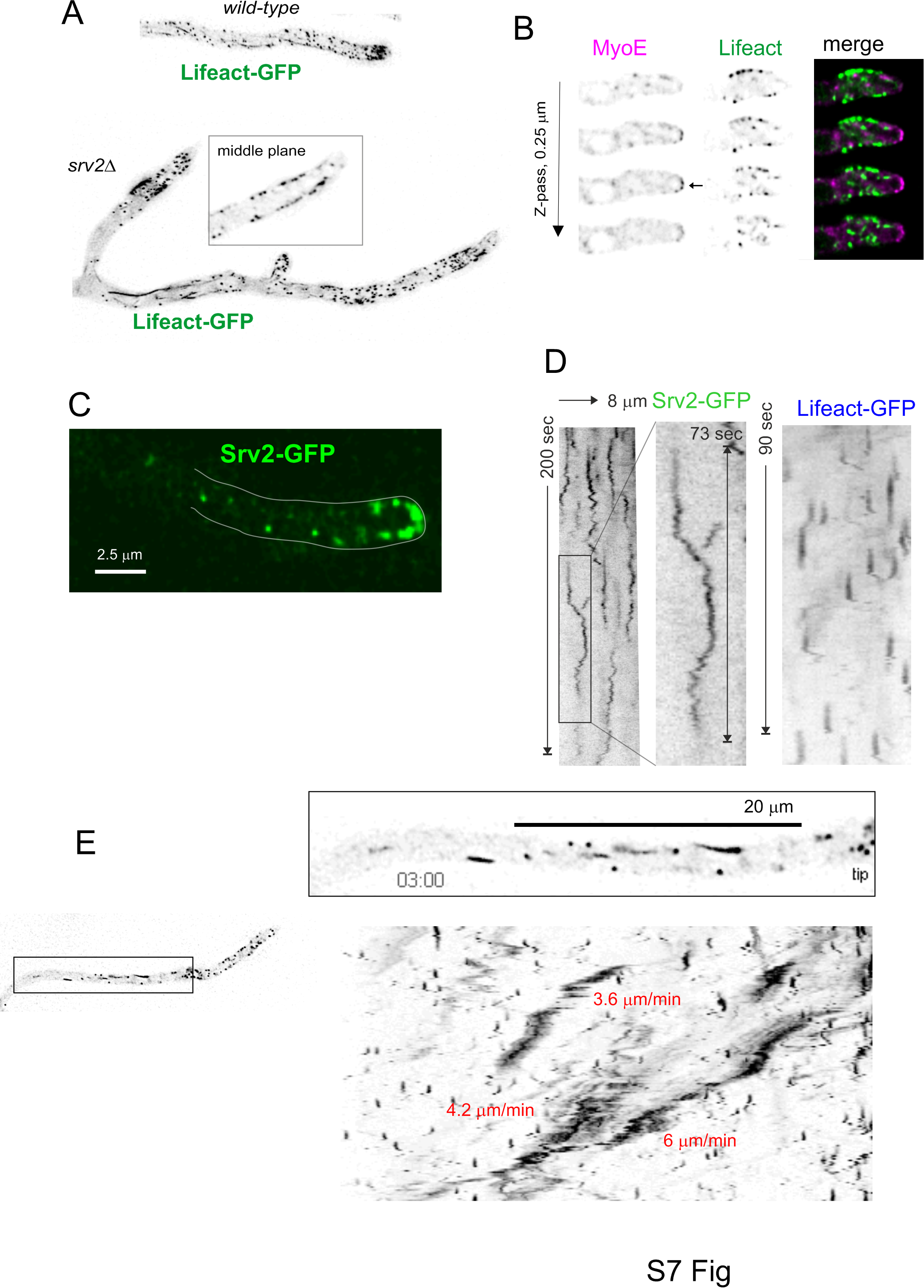

Supplement: S7 Fig — (A) Ablation of Srv2 results in disorganization of the actin collar. (B) The SPK is disorganized in srv2∆ cells. MyoE- mChFP and Lifeact-GFP distribution. MyoE forms a crescent in which individual dots can be resolved. Images are successive planes of z-stack separated by 0.25 µm in the axial dimension. (C) Localization of endogenously tagged Srv2-GFP. Note that the protein is not fully functional. (D) Lifetime of actin patches of endogenously tagged Srv2-GFP and comparison with Lifeact-GFP patches in the wild-type. (E) “Worms” of F-actin moving towards the base of an srv2Δ strain, with a kymograph showing the corresponding speeds. (TIF) [file pgen.1011619.s007.tif]

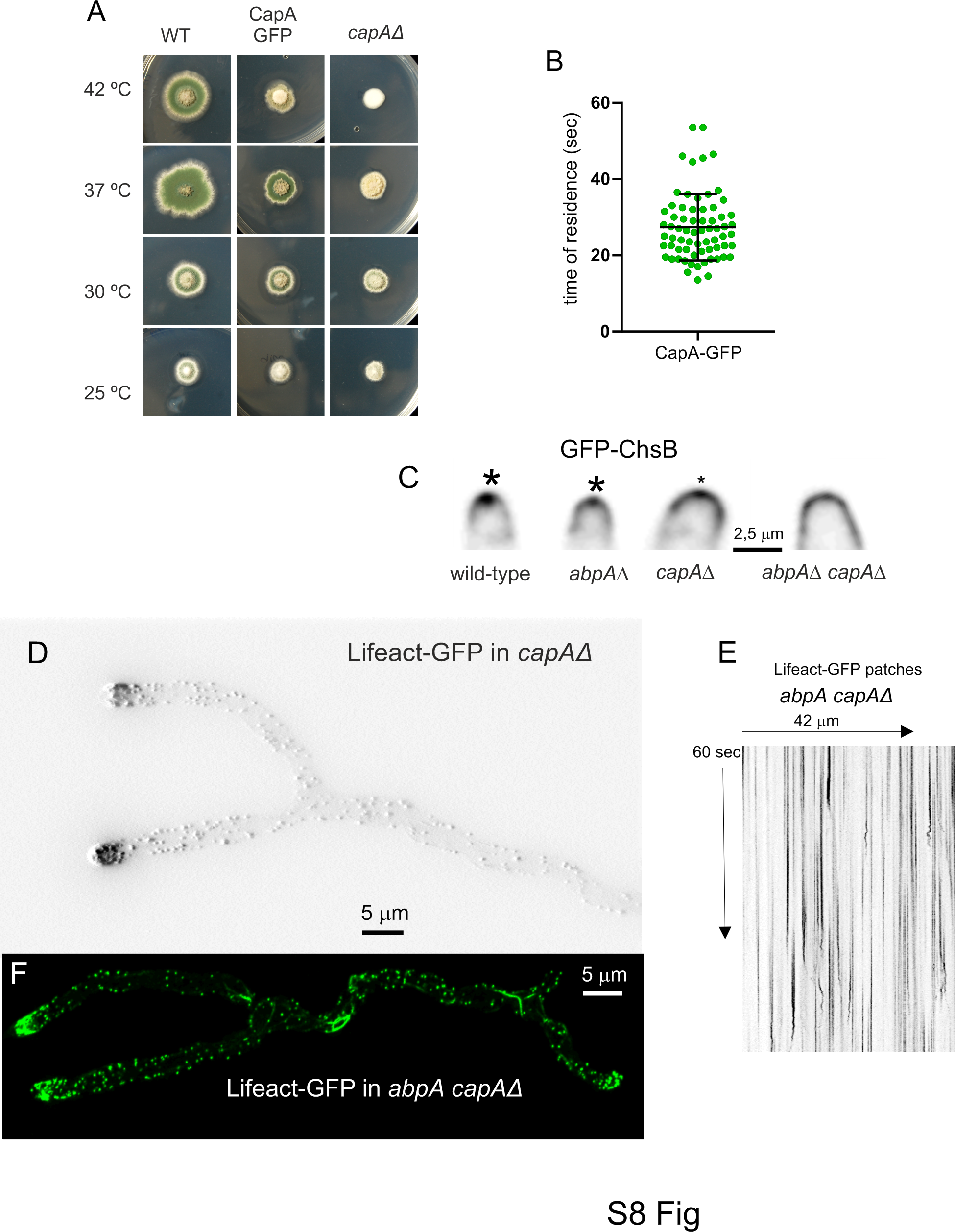

Supplement: S8 Fig — (A) Growth tests on minimal medium comparing the wild-type with a strain expressing endogenously tagged CapA-GFP and the corresponding capAΔ deletion mutant, showing that CapA-GFP is only partially functional. (B) Average lifetime of CapA-GFP in cortical patches. Error bars represent the mean ± S.D. (C) Middle planes of hyphal tips with the indicated genotypes showing the localization of the recycling cargo GFP-ChsB. Asterisks indicate the SPK, which is markedly less prominent when CapA was ablated, as indicated with a smaller asterisk. The SPK is not detectable in the abpA∆ capA∆ double mutant. (D) Split tip in a capA∆ strain expressing Lifeact-GFP. (E) Kymograph of Lifeact-GFP in the abpA∆ capA∆ double mutant showing that actin patches are static and do not mature. (F) Multiple polarity axes in an abpA∆ capA∆ double mutant cell expressing Lifeact-GFP. (TIF) [file pgen.1011619.s008.tif]

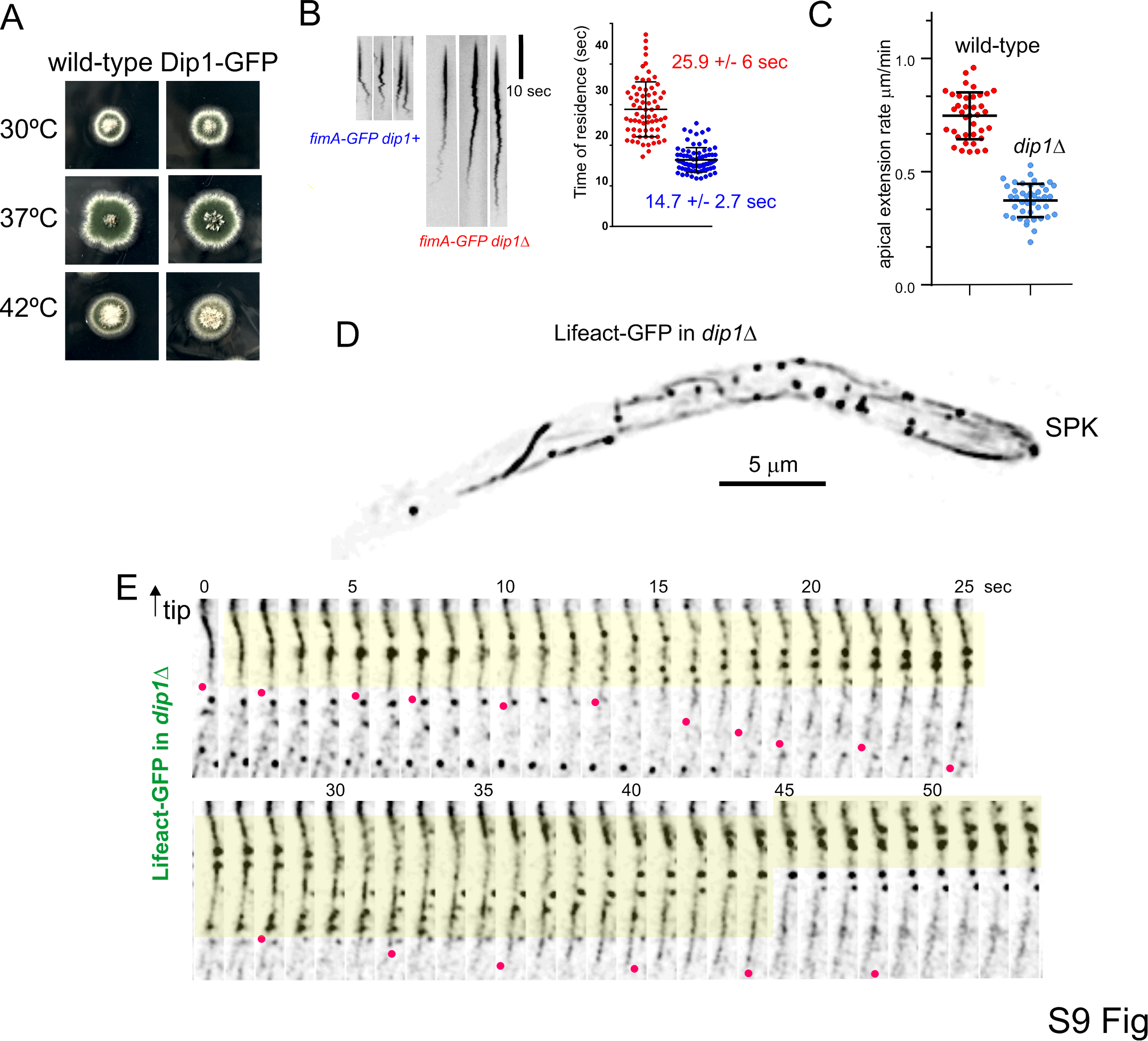

Supplement: S9 Fig — (A) Growth test showing that Dip1-GFP is functional. (B) The time of residence of GFP-tagged fimbrin in endocytic patches is nearly duplicated in dip1Δ cells, closely resembling the behavior of Lifeact-GFP. (C) Comparison of apical extension rates of the wild-type and of the dip1Δ mutant. The apical extension rate of dip1Δ tips is half of the wild-type. Error bars represent the means ± S.D. (D) Deconvolved middle plane of a dip1∆ hypha expressing Lifeact-GFP. (E) Example of an actin filament elongating throughout the sequence of 53 seconds (its leading end is indicated with a red dot). Some actin patches appeared to develop associated with the filament, whereas other appear to be captured by the growing end. (TIF) [file pgen.1011619.s009.tif]

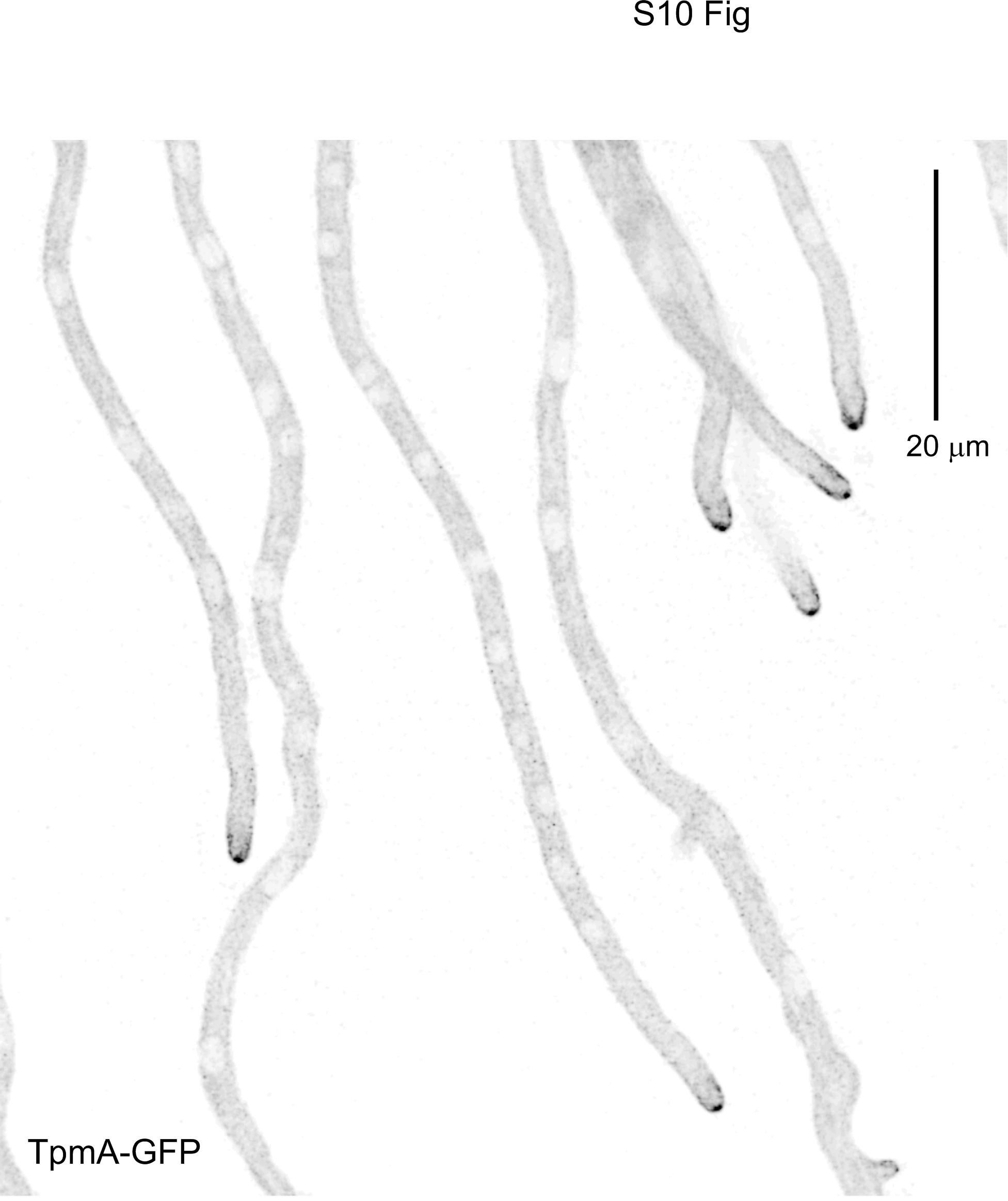

Supplement: S10 Fig — (TIF) [file pgen.1011619.s010.tif]
