# Supplementary material for: The actin module of endocytic internalization in Aspergillus nidulans: A critical role of the WISH/DIP/SPIN90 family protein Dip1
Source: PLoS Genet. 2025 Aug 26;21(8):e1011619. doi: 10.1371/journal.pgen.1011619 (PMC12422587; doi:10.1371/journal.pgen.1011619)
Supplement: S1 Table — (PDF) [file pgen.1011619.s011.pdf]

Table S1: Strains used in this work

Strain code	Genotipe
MAD0002	*wt*
MAD1420	*yA*2 *pabaA*1 *pyrG*89 *abpA*::*gfp*::*pyrG*^{Af}
MAD1750	*pyrG*89; *wA*3; *pyroA*4 *tpmA*::gfp::*pyr*4
MAD1794	*pyrG*89; *argB*2; *pyroA*4 *nkuA*Δ::*argB; slaB*::*gfp*::*pyrG*^{Af}
MAD1802	*pyrG*89; *argB*2; *pyroA*4 *nkuA*Δ::*argB*; *abpA*Δ::*pyrG*^{Af}
MAD2294	*pabaA*1 *pyrG*89?; *abpA*::*rfp*::*pyrG*^{Af}
MAD4251	*fimA*::*Tn341*::*pyr*4 *pyrG*89?; *pyroA*4 *nkuA*Δ::*bar*?
MAD5321	*pyroA*4 *nkuA*Δ::*bar*; *riboB*2
MAD5851	*pyrG*89; *nkuA*Δ::*bar pyroA*4; *pyrG*^{Af}::*gfp*::*chsB*
MAD7134	*pabaA*1; *inuAp*::*lifeact*::*gfp*::*riboB*^{Af}::*inu*At; *nku*AΔ::*bar*; *ribo*B2
MAD7183	*pyrG89; wA*::*tpmAp*::*tpmA*::*tdtomato*::*pyrG*^{Af}::*wA; pyroA*4 *nkuA*Δ::*bar*
MAD7209	*pabaA*1; *inuA*p::*lifeact*::*tdtomato*::*riboB*^{Af}::*inuA*t; *nkuA*Δ::*bar*, *riboB*2
MAD7259	*pabaA*1; *inuA*p::*lifeact*::*gfp*::*riboB*^{Af}::*inuAt*; *nkuA*Δ::*bar*?; *riboB*2; *hhoA*::rfp::*riboB*^{Af}
MAD7305	*inuA*p::*lifeact*::*tdtomato*::*riboB*^{Af}::*inuA*t; *nkuA*Δ::*bar*?; *slaB*::*gfp*::*pyrG*^{Af}
MAD7692	*pabaA*1; *wA*::*gfp*::*tubA*::*pyrG*^{Af}::*wA*; *inuA*p::*lifeact*::*tdtomato*::*riboB*^{Af}::*inuA*t; *nkuA*Δ::*bar*?
MAD7725	*inuA*p::*lifeact*::*gfp*::*riboB*^{Af}::*inuAt*; *pyroA*4 *nkuA*Δ::*bar*?; *slaB* 5'UTR::*pyrG*^{Af}::*niiA*p::*slaB*
MAD7727	*fimA*::*Tn341*::*pyr4*; *inuA*p::*lifeact*::*gfp*::*riboB*^{Af}::*inuAt*; *pyroA*4 *nkuA*Δ::*bar*?
MAD7729	*inuA*p::*lifeact*::*gfp*::*riboB*^{Af}::*inuAt*; *pyroA*4 *nkuA*Δ::*bar*?; *abpA*Δ::*pyrG*^{Af}
MAD7782	*pyrG89 yA2; argB2*::[*argB**::*alcA*p::*mCherry*::*rabA*]; *nkuA*Δ::*argB*; *pantoB*100 *capA*Δ::*pyrG*^{Af}
MAD7815	*fimA*Δ::*pyrG*^{Af} *pyrG*89; *inuA*p::*lifeact*::*gfp*::*riboB*^{Af}::*inuA*t; *pyroA*4 *nkuA*Δ::*bar*, *riboB*2
MAD7820	*yA*2 *pyrG*89?; *inuA*p::*lifeact*::*gfp*::*riboB*^{Af}::*inuAt*; *nkuA*Δ::*bar*?; *pantoB*100 *capA*Δ::*pyrG*^{Af}; *riboB*2?
MAD7965	*pyrG*89; *pyroA*4 *nkuA*Δ::*bar*; *srv2*Δ::*pyrG*^{Af}
MAD7967	*pyrG*89; *pyroA*4 *nkuA*Δ::*bar*; *srv2*::*gfp*::*pyrG*^{Af}
MAD8044	*pyrG*89 *pabaA*1; *myoE*-3x*gfp*::*pyrG*^{Af}; *nkuA*Δ::*bar*
MAD8062	*pyrG*89? *pabaA*1*; inuA*p::*lifeact*::*gfp*::*riboB*^{Af}::*InuA*t*; nkuA*Δ::*bar; srv2*Δ::*pyrG*^{Af} *riboB2*
MAD8082	*pyrG89; arpC1*::*gfp*::*pyrG*^{Af}; *pyroA*4 *nkuA*Δ::*bar*
MAD8125	*pyrG89 fimA*::*gfp*::*riboB*^{Af}*; pyroA*4 *nkuA*Δ::*bar; riboB2*
MAD8176	*pyrG*89?; *inuA*p::*lifeact*::*tdtomato*::*riboB*^{Af}::*inuA*t; *pyroA*4 *nkuA*Δ::*bar*, *arpC1*::*gfp*::*pyrG*^{Af}; *riboB*2?
MAD8181	*pyrG*89; *inuA*p::*gfp*::*actin*::*pyrG*^{Af}::*inuAt; pyroA*4 *nkuA*Δ::*bar*
MAD8184	*pyrG*89 *fimA*::*gfp*::*riboB*^{Af}; *pyroA*4 *nkuA*Δ::*bar*, *myoE*::*mcherry*::*pyrG*^{Af}; *riboB*2
MAD8186	*pyrG*89?; *myoE*::*mcherry*::*pyrGAf*; *nkuA*Δ::*bar*, *arpC1*::*gfp*::*pyrGAf*
MAD8301	*pabaA*1; *inuA*p::*actin-chromobody*::*tag-gfp*::*riboB*^{Af}::*inuA*t*; nkuA*Δ::*bar; riboB*2
MAD8303	*pabaA*1; *inuA*p::*actin-chromobody*::*mcherry*::*riboB*^{Af}::*inuA*t*; nkuA*Δ::*bar; riboB*2
MAD8559	*pabaA*1 *pyrG*89; *nkuA*Δ::*bar*, *cof1*::*gfp*::*pyrG*^{Af}/*cof1+*
MAD8641	*pyrG*89 *pabaA*1; *dip1*Δ::*pyrG*^{Af}*; nkuA*Δ::*bar*
MAD8643	*dip1*Δ::*pyrG*^{Af} *fimA*::*gfp*::*riboB*^{Af} *pyrG*89; *pyroA*4 *nkuA*Δ::*bar*, *riboB*2
MAD8726	*dip1*Δ::*pyrG*^{Af} *pyrG*89 *pabA*1; *nkuA*Δ::*bar*, *gfp*::*chsB*::*pyrG*^{Af}
MAD8728	*sepA*::3x*gfp*::*pyrG*^{Af} *pyrG*89 pabaA1; *myoE*::*mCherry*::*pyrG*^{Af}; *pyroA*4 *nkuA*Δ::*bar*
MAD8736	*dip1*Δ::*pyrG*^{Af} *sepA*::3x*gfp*::*pyrG*^{Af} *pyrG*89; *pyroA*4 *nkuA*Δ::*bar*
MAD8761	*dip1*::*gfp*::*pyrG*^{Af} *pyrG*89 *pabaA*1; *nkuA*Δ::*bar*
MAD8809	*pyrG*89; *argB*2; *pyroA*4 *nkuA*Δ::*argB*; *abpA*Δ::*pyrG*^{Af} *capA*Δ::*pyrG*^{Af} *pantoB*100
MAD8810	*pyrG*89; *pyroA*4 *nkuA*Δ::*argB*; *capA*Δ::*pyrG*^{Af}
MAD8872	*pyrG*89? *sepA*1; *myoE*-3x*gfp*::*pyrG*^{Af}; *pyroA*4 *nkuA*Δ::*bar*?
MAD8877	*inuA*p::*lifeact*::*gfp*::*riboB*^{Af}::*inuAt* *nkuA*Δ::*argB*?; *abpA*Δ::*pyrG*^{Af} *capA*Δ::*pyrG*^{Af}; *riboB*2?
MAD8917	*sepA*1; *inuA*p::*lifeact*::*gfp*::*riboBAf*::*inuA*t; *nkuA*Δ::*bar*?; *riboB2*?
MAD8939	*sepA*1 *fimA*::*mcherry*::*pyrG*^{Af} *pyrG*89?; *inuA*p::*lifeact*::*gfp*::*riboB*^{Af}::*InuA*t; *nkuA*Δ::*bar*? *riboB*2?
MAD8940	*fimA*::*mcherry*::*pyrG*^{Af} *pabaA*1 *pyrG*89?; *nkuA*Δ::*bar*?; *riboB*2?
MAD9025	*pabaA*1 *pyrG89*?; *pyroA*4 *nkuA*Δ::*bar*?; *pyrG*^{Af}::*gfp*::*chsB*; *srv2*Δ::*pyrG*^{Af}

Strain code	Genotipe
MAD9049	pyrG89; pyroA4 nkuAΔ::bar; vpr1Δ::pyrG^{Af}
MAD9052	pyrG89; pyroA4 nkuAΔ::bar?; capAΔ::pyrG^{Af} pyrG^{Af}::gfp::chsB
MAD9077	pyrG89; pyroA4 nkuAΔ::bar; vpr1::gfp::pyrG^{Af}
MAD9080	pyrG89; inuAp::tractin-gfp::pyrG^{Af}::inuAt; pyroA4 nkuAΔ::bar
MAD9083	dip1Δ::pyrG^{Af} pyrG89 pabaA1; inuAp::lifeact::gfp::riboB^{Af}::inuAt; nkuAΔ::bar; riboB2
MAD9085	pyrG89 pabaA1; inuAp::lifeact::gfp::riboB^{Af}::inuAt; nkuAΔ::bar; riboB2 vpr1Δ::pyrG^{Af}
MAD9089	pyrG89; nkuAΔ::bar pyroA4; riboB^{Af}::gfp::chsB; vpr1Δ::pyrG^{Af} riboB2
MAD9092	pyrG89; pyroA4 nkuAΔ::bar?; abpAΔ::pyrG^{Af} pyrG^{Af}::gfp::chsB
MAD9094	pyrG89; pyroA4 nkuAΔ::bar?; capAΔ::pyrG^{Af} abpAΔ::pyrG^{Af} pyrG^{Af}::gfp::chsB
MAD9100	pyrG89; pyroA4 nkuAΔ::bar; capA::gfp:pyrG^{Af}
MAD9296	pyrG89; pyroA4 nkuAΔ::bar; arpC1::mcherry::pyroA^{Af}; vpr1::gfp::pyrG^{Af}
MAD9369	pyrG89; myoE::mcherry::pyrG^{Af} inuAp::lifeact::gfp::riboB^{Af}::inuAt; nkuAΔ::bar; riboB2?
MAD9400	sepA1 pyrG89? pabaA1; dip1Δ::pyrG^{Af}; inuAp::lifeact::gfp::riboB^{Af}::inuAt; nkuAΔ::bar?; riboB2?
MAD9402	pyrG89?; myoE-mcherry::pyrG^{Af} inuAp::lifeact::gfp::riboB^{Af}::inuAt; nkuAΔ::bar?; abpAΔ::pyrG^{Af} pantoB100
MAD9404	pyrG89?; myoE-mcherry::pyrG^{Af} inuAp::lifeact::gfp::riboB^{Af}::inuAt; nkuAΔ::bar?; srv2Δ::pyrG^{Af}
MAD9407	pyrG89?; myoE-mCherry::pyrG^{Af} inuAp::lifeact::gfp::riboB^{Af}::inuAt; nkuAΔ::bar?; capAΔ::pyrG^{Af} pantoB100
MAD9409	pyrG89?; myoE-mcherry::pyrG^{Af} inuAp::lifeact::gfp::riboB^{Af}::inuAt; pyroA4 nkuAΔ::bar?; capAΔ::pyrG^{Af} abpAΔ::pyrG^{Af}; pantoB100
MAD9412	pyrG89 pabaA1; inuAp::lifeact::Tdtomato::riboB^{Af}::inuAt; nkuAΔ::bar; riboB2 end3::gfp::pyrG^{Af}
MAD9414	pyrG89; nkuAΔ::bar pyroA4; end3::gfp::pyrG^{Af}
MAD9416	fimA::Tn341::pyr4; myoE::mcherry::pyrG^{Af} inuAp::lifeact::gfp::riboB^{Af}::inuAt; nkuAΔ::bar?
MAD9423	fimA::Tn341::pyr4 pyrG89?; synA::gfp::pyrG^{Af}; pyroA4 nkuAΔ::bar?
