# Supplementary material for: The actin module of endocytic internalization in Aspergillus nidulans: A critical role of the WISH/DIP/SPIN90 family protein Dip1
Source: PLoS Genet. 2025 Aug 26;21(8):e1011619. doi: 10.1371/journal.pgen.1011619 (PMC12422587; doi:10.1371/journal.pgen.1011619)
Supplement: S2 Table — (PDF) [file pgen.1011619.s012.pdf]

Supplemental Table 2. Oligonucleotides used for genetic manipulation and plasmids constructions.

**Primer name**	**Sequence 5´-3´**
**pGEM-T *inuAp*::*lifeact*::*gfp*::*riboBAf*::*inuA*t / *pGEM-T inuAp*::*lifeact*::*tdtomato*::*riboBAf*::*inuA*t**	
Fw inuAp	GTGGAGGCCACTCTCGGAAAC
Rev InuAp join lifeact	CTCCTCCTTGGAGATGGACTCGAACTTCTTGATGAGGTCGGCGACGCCCATTTTGGTGATGTCGCTGACCGC
Fw GA5 join lifeact	ATGGGCGTCGCCGACCTCATCAAGAAGTTCGAGTCCATCTCCAAGGAGGAGGGAGCTGGTGCAGGCGCTGGAG
Fw inuAt join riboBAf	ACACGGTGTATGCTCGTCACACTCATAGGATCTAGCTAGATGTTTTGTTG
Rv RiboBAf	ATGAGTGTGACGAGCATACACC
Rv inuAt	CAGCAGTCAAGCAATACCAAGC
***inuA*p::*Actin-Chromobody*::*tag-gfp*::*riboBAf*::*inuA*t DNA cassette**	
Fw inuAp	GTGGAGGCCACTCTCGGAAAC
Rv inuAp join Actin-Chr-TagGFP	CAGACTCCACCAGCTGCACCTGAGCCATTTTGGTGATGTCGCTGACCGC
Fw Actin-Chr tagGFP join InuAp	TACGCGCGGTCAGCGACATCACCAAAATGGCTCAGGTGCAGCTGGTG
Fw RiboBAf join Actin-Chr-TagGFP	GCATGGACGAGCTGTACAGGTAAAAGAGGCCGTTCAGGAGTCTGG
Rv Tag-GFP join RiboBAf	CGAGCCAGACTCCTGAACGGCCTCTTTTACCTGTACAGCTCGTCCATGC
Rv RiboBAf join InuAt	CAACAAAACATCTAGCTAGATCCTCAGAACGTTTGCGCTGCAGAACCG
Fw InuAt join RiboBAf	GGTTCTGCAGCGCAAACGTTCTGAGGATCTAGCTAGATGTTTTGTTG
Rv InuAt	CAGCAGTCAAGCAATACCAAGC
***inuA*p::*Actin-Chromobody*::*mCherry*::*riboBAf*::*inuA*t DNA cassette**	
Fw inuAp	GTGGAGGCCACTCTCGGAAAC
Rv inuAp join Actin-Chr	CAGACTCCACCAGCTGCACCTGAGCCATTTTGGTGATGTCGCTGACCGC
Fw Actin-Chr join InuAp	TACGCGCGGTCAGCGACATCACCAAAATGGCTCAGGTGCAGCTGGTG
Rv Actin chr join mCherry	CGGCTCCAGCGCCTGCACCAGCTCCTGAGGAGACGGTGACCTGGGTC
Fw mChery join Actin Chr	GAGGGGACCCAGGTCACCGTCTCCTCAGGAGCTGGTGCAGGCGCTGGAG
Rv mCherry join RiboBAf	GCGGCATGGACGAGCTGTACAAGTAAAAGAGGCCGTTCAGGAGTCTGG
Rv RiboBAf join InuAt	CAACAAAACATCTAGCTAGATCCTCAGAACGTTTGCGCTGCAGAACCG
Fw InuAt join RiboBAf	GGTTCTGCAGCGCAAACGTTCTGAGGATCTAGCTAGATGTTTTGTTG
Rv InuAt	CAGCAGTCAAGCAATACCAAGC
***InuA*p::*Tractin*::*gfp*::*pyrGAf*::*inuA*t DNA cassette**	
Fw inuAp	GTGGAGGCCACTCTCGGAAAC
Rv Tractin join Gas-GFP::PyrGAf	CACCGGCTCCAGCGCCTGCACCAGCTCCGCCGGCAGCGGCAGCGGCAGC
Fw GAs-GFP::PyrGAf join Tractin	GCCGTCGCTGCCGCTGCCGCTGCCGGCGGAGCTGGTGCAGGCGCTGGAG
Rv PyrGaf join InuAt	AACAACAAAACATCTAGCTAGATCCTGTCTGAGAGGAGGCACTGATG
Fw inuAt join PyrGaf	ACGCATCAGTGCCTCCTCTCAGACAGGATCTAGCTAGATGTTTTGTTG
Rv InuAt	CAGCAGTCAAGCAATACCAAGC
***inuA*p::*gfp*::*Actin*::*pyrGAf*::*inuA*t DNA cassette**	
Fw inuAp	GTGGAGGCCACTCTCGGAAAC
Rv inuAp join GFP	CAGTGAAAAGTTCTTCTCCTTTACTCATTTTGGTGATGTCGCTGACCGC
Fw GFP join InuAp	CGCGCGGTCAGCGACATCACCAAAATGAGTAAAGGAGAAGAACTTTTC
Rv GAs GFP join Actin	GATAACGAGAGCAGCAACTTCCTCTTCCATGGCACCGGCTCCAGCGCCTG

Fw Actin join GAs GFP	CTGGTGCAGGCGCTGGAGCCGGTGCCATGGAAGAGGAAGTTGCTGCTC
Rv Actin Join PyrGAf	TCCAGCACACTGGCGGCCGTTACCTTGTATAACTAGCAATACTGTACTA
Fw pyrGAf Join Actin	TAGTACAGTATTGCTAGTTATACAAGGTAACGGCCGCCAGTGTGCTG
Rv PyrGaf join InuAt	AACAACAAAACATCTAGCTAGATCCTGTCTGAGAGGAGGCACTGATG
Fw inuAt join PyrGaf	ACGCATCAGTGCCTCCTCTCAGACAGGATCTAGCTAGATGTTTTGTTG
Rv InuAt	CAGCAGTCAAGCAATACCAAGC

***TpmA*::*tdTomato* cassette**

Fw wA 5´region	CGTCTATTGTAGAGCCTCCATGC
Rv wA 5´region join TpmA	GACATCTACTCGCTCTTGGATGACAAGTGATCAGGAGAAGGAGAGTCAAG
Fw TpmA Join wA 5´region	GCGGACTTGACTCTCCTTCTCCTGATCACTTGTCATCCAAGAGCGAGTAG
Rv TpmA join tdT::pyrGAf	CTATTCTAGGCGCGCCAACACTGTTCAAGGAGAGCTCG
Rv tdT::pyrGAf join wA 3´	GAAACGGGAAAGTAAAACCTTATGAGCGTCTGAGAGGAGGCACTGATG
Fw wA 3´region join pyrGAf	TCACGCATCAGTGCCTCCTCTCAGACGCTCATAAGGTTTTACTTTCCCG
Rv wA 3´region	CACCTGACTCGCATTGGAGAAC

***ArpC1::gfp* cassette**

Fw ORF *ARPC1*	CAGGCTCAACTGACTCTCATGC
Rev ORF *RPC1*	GATGGTCCAGATGACGACACGG
Fw join ORF *ARPC1 GFP-PyrG^{Af}*	GACGGCCGTGTCGTCATCTGGACCATCGGAGCTGGTGCAGGCGCTGGAG
Rv join 3UTR *ARPC1-pyrG^{Af}*	GTATGAAGCTCAGCCCAAGAACCAACGTCTGAGAGGAGGCACTGATG
Fw 3UTR *ARPC1*	GTTGGTTCTTGGGCTGAGCTTCAT
Rv 3UTR *ARPC1*	GGTGCATCGCCGTCAGTATCG

***ArpC1* Knock- Out cassette**

Fw 5UTR *ARPC1*	CCAGAAGGCTTTGGAGGCAAAGC
Rev 5UTR *ARPC1*	GTTGTTGTAAGGACAGGAGGAG
Fw join 5UTR *ARPC1-pyrG^{Af}*	GAGAGCTCCTCCTGTCCTTACAACAACGTAACGGCCGCCAGTGTGCTGG
Rv join 3UTR *ARPC1-pyrG^{Af}*	GTATGAAGCTCAGCCCAAGAACCAACGTCTGAGAGGAGGCACTGATG
Fw 3UTR *ARPC1*	GTTGGTTCTTGGGCTGAGCTTCAT
Rv 3UTR *ARPC1*	GGTGCATCGCCGTCAGTATCG

***sepA*::3x*gfp* cassette**

Fw *sepA* ORF	TTGGCTGCGTTCTTACAGGAG
Rv sepA ORF join GAs GFPs	CCGGCTCCAGCGCCTGCACCAGCTCCGCTGGTCGAGGGGCGATGTTC
Fw GAs GFPs join SepA	GATGATGAACATCGCCCCTCGACCAGCGGAGCTGGTGCAGGCGCTGGAG
Rv GFP join PyrGaf	CTTGTAGAGCTCGTCCATGCC
Fw PyrGAf	GCCTCAAACAATGCTCTTCACC
Rv PyrGAf join 3 UTR sepA	GGTAATAGGTCGGTGTGTGCGTCGGTCTGAGAGGAGGCACTGATG
Fw 3 UTR sepA join pyrGAf	CACGCATCAGTGCCTCCTCTCAGACCGACGCACACACCGACCTATTACC
Rv 3 UTR sepA	CTGCGAAGCATCGATGTTG

***Vpr1* Knock- Out cassette**

Fw 5UTR *VPR1*	GCGTGTGGACAATGCGGCAGAG
Rev 5UTR *VPR1*	GAGGGATCAGTAGAGATAGGG
Fw join 5UTR *VPR1-pyrG^{Af}*	ACCACGTCCCTATCTCTACTGATCCCTCGTAACGGCCGCCAGTGTGCTG
Rv join 3UTR *VPR1-pyrG^{Af}*	TTGTGGGTACAGTATCAATGCTAATAGGTCTGAGAGGAGGCACTGATG
Fw 3UTR *VPR1*	CTATTAGCATTGATACTGTACCC
Rv 3UTR *VPR1*	CAATTGCTTCATCCACTTCACC

***Vpr1::gfp* cassette**

Fw ORF *VPR1*	GTCGAGGAGGTGTTGATACTGG
Rev ORF *VPR1*	GCCGCTTAACGCGCTGAGATCC
Fw join ORF *VPR1 GFP-* $PyrG^{Af}$	GCCGTTGGATCTCAGCGCGTTAAGCGGCGGAGCTGGTGCAGGCGCTGGAG
Rv join 3UTR *VPR1-* $pyrG^{Af}$	TTGTGGGTACAGTATCAATGCTAATAGGTCTGAGAGGAGGCACTGATG
Fw 3UT *VPR1*	CTATTAGCATTGATACTGTACCC
Rv 3UTR *VPR1*	CAATTGCTTCATCCACTTCACC

***FimA* Knock- Out cassette**

Fw 5UTR *FIMA*	CGTTGTTACCGGACGCGGCTC
Rev 5UTR *FIMA*	CAGAATTTGGCTCCCCTCTCGC
Fw join 5UTR *FIMA -* $pyrG^{Af}$	GCCATTGCGAGAGGGGAGCCAAATTCTGGTAACGGCCGCCAGTGTGCTG
Rv join 3UTR *FIMA -* $pyrG^{Af}$	GTTCACAGGCTGCAGCCGTGAAGCGTCTGAGAGGAGGCACTGATG
Fw 3UTR *FIMA*	GCTTCACGGCTGCAGCCTGTG
Rv 3UTR *FIMA*	CATAGCAGCGGGAACGAGTGC

***Srv2* Knock- Out cassette**

Fw 5UTR *SRV2*	CGGTCCGTAACCATTCCATGC
Rev 5UTR *SRV2*	CTTGTCGGCTTTCTGGGCAGC
Fw join 5UTR *SRV2 -* $pyrG^{Af}$	GCCTCTGCTGCCCAGAAAGCCGACAAGGTAACGGCCGCCAGTGTGCTG
Rv join 3UTR *SRV2 -* $pyrG^{Af}$	AGGACAGGCCCAAAGTTAACGCCTGTGGTCTGAGAGGAGGCACTGATG
Fw 3UTR *SRV2*	CACAGGCGTTAACTTTGGGCCTGTC
Rv 3UTR *SRV2*	GTTGCCTCATACTAGCATAGAGATG

***Srv2::gfp* Cassette**

Fw OR *SRV2*	GGATGGAATAGATGCACAGGAGG
Rev ORF *SRV2*	GCCCGCATGCTCGACAATTTCG
Fw join ORF *SRV2 GFP-* $PyrG^{Af}$	GTCAGCGAAATTGTCGAGCATGCGGGCGGAGCTGGTGCAGGCGCTGGAG
Rv join 3UTR *SRV2 -* $pyrG^{Af}$	AGGACAGGCCCAAAGTTAACGCCTGTGGTCTGAGAGGAGGCACTGATG
Fw 3UTR *SRV2*	CACAGGCGTTAACTTTGGGCCTGTC
Rv 3UTR *SRV2*	GTTGCCTCATACTAGCATAGAGATG

***Dip1* Knock- Out cassette**

Fw 5UTR *Dip1*	GATGTTCCAGGCTGTCGACAAAG
Rev 5UTR *Dip1*	CCCACAACTCGGTCGTTGTCC
Fw join 5UTR *Dip1 -* $pyrG^{Af}$	GGCCCGAGGACAACGACCGAGTTGTGGGGTAACGGCCGCCAGTGTGCTG
Rv join 3UTR *Dip1 -* $pyrG^{Af}$	TGCCTCGACCAAACCCGCTCGTTTCGGGTCTGAGAGGAGGCACTGATG
Fw 3UTR *Dip1*	CCGAAACGAGCGGGTTTGGTC
Rv 3UTR *Dip1*	CAGTGAGACGCCATCAGTTACC

***Dip1::gfp* cassette**

Fw ORf *Dip1*	CAACAGTAGACGAAACCGCCTC
Rev ORF *Dip1*	GTGCAGCGAGACCTTCTGCAAAG
Fw join ORF *Dip1 GFP-* $PyrG^{Af}$	GAGGCTTTGCAGAAGGTCTCGCTGCACGGAGCTGGTGCAGGCGCTGGAG
Rv join 3UTR *Dip1 -* $pyrG^{Af}$	TGCCTCGACCAAACCCGCTCGTTTCGGGTCTGAGAGGAGGCACTGATG
Fw 3UTR *Dip1*	CCGAAACGAGCGGGTTTGGTC
Rv 3UTR *Dip1*	CAGTGAGACGCCATCAGTTACC

***CapA::gfp* cassette**

Fw ORF *CapA*	GAGGTGGGTTGTCGGTTCTACG

Rev ORF *CapA*	CCGCCCCTTTCCACCAGAGATATC
Fw join ORF *CapA GFP-PyrG*^{Af}	GGATATCTCTGGTGGAAAGGGGCGGGGAGCTGGTGCAGGCGCTGGAG
Rv join 3UTR *CapA -pyrG*^{Af}	CACAGAGAAAAACCACTGAGGTCCAGCGTCTGAGAGGAGGCACTGATGC
Fw 3UTR *CapA*	GCTGGACCTCAGTGGTTTTTCTCTG
Rv 3UTR *CapA*	GAGTCTGGTCAGGATACCGGAG

***Cof1* Knock- Out cassette**

Fw 5UTR *Cof1*	CTACTGGCCTTACCACTGTTTGC
Rev 5UTR *Cof1*	GCAATATGTGATTTGCACTGGCAG
Fw join 5UTR *Cof1 -pyrG*^{Af}	GTCGTCTGCCAGTGCAAATCACATATTGCGTAACGGCCGCCAGTGTGCTG
Rv join 3UTR *Cof1 -pyrG*^{Af}	GGCCCGGACCCAGCCGTCGCCATTTGGTCTGAGAGGAGGCACTGATG
Fw 3UTR *Cof1*	CAAATGGCGACGGCTGGGTCC
Rv 3UTR *Cof1*	CTAGAGATAGTGCGGTAAGAGC

***Cof1::gfp* cassette**

Fw ORF *Cof1*	CGCATACATTGGAAATTCGTTCTG
Rev ORF *Cof1*	TTTGGCCTTACCACCGCTGACC
Fw join ORF *Cof1 GFP-PyrG*^{Af}	GCCGAGGTCAGCGGTGGTAAGGCCAAAGGAGCTGGTGCAGGCGCTGGAG
Rv join 3UTR *Cof1 -pyrG*^{Af}	GGCCCGGACCCAGCCGTCGCCATTTGGTCTGAGAGGAGGCACTGATG
Fw 3UTR *Cof1*	CAAATGGCGACGGCTGGGTCC
Rv 3UTR *Cof1*	CTAGAGATAGTGCGGTAAGAGC

***FimA*::*mCherry* cassette**

Fw ORF *FimA*	CAATGGCCGCGAAGGTAAGGCACC
Rev ORF*FimA*	CATTTTTTCGTACGTAGCCATTAGA
Fw join ORF *FimA* mCherry	TCTCTAATGGCTACGTACGAAAAAATGGGAGCTGGTGCAGGCGCTGGAG
Fw mCherry join *pyrG*^{Af}	GCGGCATGGACGAGCTGTACAAGTAAGTAACGGCCGCCAGTGTGCTG
Rv mCherry join *pyrG*^{Af}	GAATTCCAGCACACTGGCGGCCGTTACTTACTTGTACAGCTCGTCCATGC
Rv join 3UTR *FimA- pyrG*^{Af}	GTTCACAGGCTGCAGCCGTGAAGCGTCTGAGAGGAGGCACTGATG
Fw 3UTR *FimA*	GCTTCACGGCTGCAGCCTGTG
Rv 3UTR *FimA*	CATAGCAGCGGGAACGAGTGC

***FimA*::*gfp* cassette**

Fw ORF *FimA*	CAATGGCCGCGAAGGTAAGGCACC
Rev ORF *FimA*	CATTTTTTCGTACGTAGCCATTAGA
Fw join ORF *FimA GFP-RiboB*^{Af}	TCTCTAATGGCTACGTACGAAAAAATGGGAGCTGGTGCAGGCGCTGGAG
Rv join 3UTR *FimA -RiboB*^{Af}	GTTCACAGGCTGCAGCCGTGAAGCATGAGTGTGACGAGCATACACCGTG
Fw 3UTR *FimA*	GCTTCACGGCTGCAGCCTGTG
Rv 3UTR *FimA*	CATAGCAGCGGGAACGAGTGC

***End3*::*gfp* cassette**

Fw ORF *End3*	GTACGTTTGTCGCTACGGCTTGGG
Rv ORF *End3*	CCGAGACGCCCGCTCGTCTTC
Fw join ORF *End3 GFP-pyrG*^{Af}	GAAATTGAAGACGAGCGGGCGTCTCGGGGAGCTGGTGCAGGCGCTGGAG
Rv join ORF *End3 GFP-pyrG*^{Af}	GCCAGGATAGGTACAAGAAGGAAGACGGTCTGAGAGGAGGCACTGATG
Fw 3UTR *End3*	CGTCTTCCTTCTTGTACCTATCC
Rv 3UTR *End3*	GACTCGGACTGGAACCCGCAG
