# Supplementary material for: The actin module of endocytic internalization in Aspergillus nidulans: A critical role of the WISH/DIP/SPIN90 family protein Dip1
Source: PLoS Genet. 2025 Aug 26;21(8):e1011619. doi: 10.1371/journal.pgen.1011619 (PMC12422587; doi:10.1371/journal.pgen.1011619)
Supplement: Attachment — Submitted filename: Response to referees R1.docx [file pgen.1011619.s043.docx]

Thank you very much for the time you have invested in revising this article and for your positive comments and sound suggestions.

The new additions and modifications of the manuscript can be summarized as follows:

Methodological details on the construction of kymographs and their interpretation have been introduced in the Materials and Methods section, as well as in the relevant paragraph of the Results section (p9-11). Additionally, we have added a supplemental movie S6Mov and a new S3 Fig to illustrate our points.

A clear discussion on our interpretation of the origin of worms and the difference between worms and exocytic cables has been added into the discussion section (see our response to referee 1). Additional experiments to clarify our points have been introduced, including. New Figs 5, Fig 15I (previously provided as supplemental), Fig 15J, S1 Fig, S9E Fig, S10 Fig, Movies S10M, S12M, S27M, S28M & S29M)

The role of formin in the biogenesis of exocytic cables and worms: We have gone as far as possible exploiting the *sepA1* heat-sensitive mutation in the formin gene. We have constructed the double mutant and concluded that formin does not impair the endocytic phenotype of *dip1∆* much further. It is clear that formin is required for the biogenesis of both worms and exocytic cables. But only with the tool of heat-sensitive mutation at hand (CK666 does not work in *Aspergillus nidulans*), all we can say is that it appears that neither worms nor exocytic cables are required for the biogenesis of patches in the *dip1∆* mutant. However, we note, and we should like to emphasize, that these experiments do not rule out the possibility that small pieces of filamentous actin resulting from the turnover of larger structures generated before shifting cells to a restricted temperature serve as seeds for dendritic polymerization, following the model of Tom Pollard. New experiments have been introduced, and figures and movies have been added. Once we have finished this new set of experiments, we realized that the model displayed on former figure 16, while still possible, was too hypothetical to be displayed as a conclusion of the work. We have therefore removed this figure in addition to using careful wording hither and thither.

Phenotyping the *sepA1 dip1∆* double mutant: this has been answered in part in the previous paragraph. We conclude that there is no hint pointing that the possible involvement of formin in the biogenesis of actin patches. The requested experiments are displayed on new figure 16, which substitutes for the former model Figure.

Another important point that required clarification is the relationship between F-actin in the SPK and the structural marker of the SPK. We have not used FM4-64, despite the fact that I pioneered its use in *A. nidulans* (Penalva 2005 PMID: 16291501). It is well established that in *A. nidulans* FM4-64 labels mitochondria very strongly (Fischer-Parton, 2000, PMID: 10849201, making it difficult to distinguish the SPK, as mitochondria very often colonize the tip region. We have invested some time in obtaining good pictures, and one of them is displayed on Figure 1E, but it is beyond doubt that FM4-64 is not a reliable marker for the detection of the SPK. Therefore, we followed the suggestion of the referee of using the type V myosin MyoE. We have now introduced experiments with MyoE and Lifeact GFP onto Figure 1F, Figure 7, Figure 9, Figure 13, Figure 16B and Figure S5, and with ChsB and SynA on Figures S8E and S5, respectively. Only in one case we have used FM 4-64, which is the case of cofilin-GFP, that we photographed in a diploid, and therefore this strain is not immediately amenable to genetic manipulation. The experiment is displayed on Figure 8D.

Finally, from the point of view of presentation, besides having removed former Figures 7 (prompted by the referees) and 16 (the model), we have introduced new figures and movies to address the comments of the referees. This revised version of the manuscript contains 16 Main figures, 10 supplemental figures and 29 movies.

Below you may find a detailed account of how we have responded to the suggestions and criticisms of the referees.

Reviewer #1: This manuscript from the Penalva lab describes endocytic actin patches in Aspergillus and covers a large ground. The authors can be commended for their extensive work, high-quality imaging, and clear descriptions, which brings high standards to cell biology work with this filamentous organism. By tagging and deleting key proteins involved in actin assembly and regulation, focusing on regulators of actin patches in yeast, they describe similarities and differences in Aspergillus compared to the well-studied yeast cases. An important focus of the work is on identifying the source of the mother filament for Arp2/3 assembly at endocytic sites. They reveal an interesting actin “worm” structure, which often overlaps with actin patches and is suggested to help new nucleation. They also describe the phenotype of dip1∆, which largely, but not completely, abrogates patch formation.

Overall, I find the work very interesting and well supported. I have two main comments – on methodology and on the dip1-independent source of mother filaments – which I feel need to be addressed before publication. These experiments should be doable with the tools that the authors have at their disposal.

**Major points:**

It is not clear to this reviewer exactly how kymographs are constructed, as this is not clearly explained in the methods. For longitudinal kymographs, used in most of the study, what is the width of the line that is captured? If the line is only drawn at the cell cortex, such kymographs cannot capture the inward movement of actin patches as they invaginate and break off from the plasma membrane, as they would simply disappear from the kymograph. However, it seems that the line becomes then wispy and wiggly, suggesting that part of the inward movement is captured. How much of it? It is also not clear whether the transition to invagination can be clearly established with this analysis strategy, in contrast to the transversal approach used in most of the yeast work. In the examples shown in Fig 3C, taking the transition from a static to a wiggly line as start of invagination, it seems like the residence time at the membrane is much shorter in the transversal than longitudinal examples. These points should be clarified to better establish the analysis method. More generally, there needs to be more precise of image quantification methods. Currently, all is stated is that “the length of the traces was estimated visually and converted into timescales”. This does not suffice for anyone to reproduce the work.

The referee is quite right, there are aspects of the methodology that were insufficiently described. The decision of using longitudinal rather than transverse kymographs was largely dictated by the fact that patches move in both directions during the wiggly phase and, in addition, move with the cell, which is growing rapidly. Therefore, capturing the whole lifecycle of a given patch using a transversal ROI requires a wide line, and this implies that the chances of another patch entering into the ROI are very high, limiting the number of events that can be analyzed. In contrast, using longitudinal kymographs, we set up linear ROIs with enough pixels to cover at least half of the hyphal width, usually the whole width. This method has an intrinsic problem, which is that considering the high density of patches per unit length, it is often difficult to identify individual patches whose kymograph traces do not overlap. In addition to choosing subapical regions to avoid the high concentration of patches near the endocytic collar, we converted X, Y, T stacks into X, Y, Z stacks that could be analyzed with a 3-D viewer, which facilitated the neat separation of traces that overlapped in 2D (revised Movie 6). This description of the methodology is briefly discussed in the results section (p9-11) and expanded in full in the materials and methods (p31). A classical montage depicting the lifecycle of one Lifeact-GFP patch is shown compared to the kymograph analysis of the same patch on Fig 3D.

An important point included in this comment is related to our taking the point at which the wiggly lines are no longer visible as the point at which the patch transits to invagination. The referee is quite right in that we haven’t formally identified the point at which the endocytic patch is actually endocytosed. The reproducibility of the patch lifecycles, the fact that the length of the actin phase is quite similar to those of the model yeasts, and the coincidence in the length of the lifecycles among different components of the actin phase are consistent with this assumption, but did not show it. We thought it would be fair to mention this explicitly in the text (first complete paragraph on p11).

As for the apparent time of residence of actin being different from that detected with transversal kymographs, the intrinsic variability between patches almost certainly accounts for these differences. We do not think this deserves a comment in the main text, but have added in the new supplemental Figure 3 a few examples that illustrate this variability

*********************************************************************
The origin of the actin will “worms” is mysterious. The text variously hypothesizes that they originate from the SPK, implying a formin-dependent assembly, or that they initiate at a patch, suggesting elongation of Arp2/3-nucleated filaments. With the tools and mutants at hand, this question should be quite easily addressed: Are the actin “worms” labeled by tropomyosin, as would be likely expected if they are composed of linear actin filaments? Do they form in the sepA mutant?

This was a very fair comment that called our attention to one aspect of the manuscript that indeed necessitated improvements. We have addressed this point all across the manuscript. It is pretty clear that worms derive from longer cables originating from actin patches, are able to move for long distances and are not decorated with tropomyosin. In contrast, exocytic actin cables originate at the SPK, are disassembled at certain distance from the tip, and are decorated by tropomyosin. This is now considered in detail in the discussion, starting with the last paragraph on page 26 up to the end of the discussion on page 28. The direct answer to the question of whether worms form in the *sepA1* mutant is that they do not. We note that interpretation of this result is not straightforward and will be a topic for future investigation.

Linked to the previous issue, the manuscript makes a strong statement that formin is not involved in generating the mother filament for Arp2/3 nucleation. However, the authors show that, although Dip1 plays an important role, there is also a Dip1-independent mechanism. It seems entirely possible to me that this Dip1-independent mechanism relies on formin activity, whose role may be masked in presence of Dip1. To test this, it would be important to examine the phenotype of dip1∆ sepA double mutants.

Thank you very much for this sound comment We have also addressed whether a hypothetical activity of formin in the generation of mother filaments is uncovered in the absence of *dip1* by constructing the *dip1∆ sepA1* double mutant. With the caveat that the *sepA1* mutation is not a null allele and might potentially have allele-specific interactions, growth of the double mutant is indistinguishable from that of the *sepA1* single mutant at 30ºC and at 37°C, the latter being a temperature in which *sepA1* behaves as strong hypomorph. The clear-cut result is that in the double mutant actin patches still form and cycle more or less normally. We appreciate the fact that we are dealing with a missense mutation and that formin plays multiple roles in the cell, but this experiment clearly adds to evidence against a putative role of formin in endocytosis. These experiments are described in new Figure 16 and movie S29.

Alternatively, the mother filament may come from another actin patch. This can also be tested by performing transient depolymerization assays with CK-666 and probing the ability of actin patches to regenerate after washout (in dip1∆, or in dip1∆ sepA∆ if the latter still shows actin patches).

This is true and in fact we have documented some examples in which this might appear to be the case. However, we note that this interpretation brings down the problem of actin patch biogenesis to the ‘mother of the mothers’ filament. In the discussion, we now considered the possibility that small actin filaments resulting from the turnover of other F-actin structures can provide the seed for new patches. This idea is interesting enough to be followed up. Unfortunately, CK-666 does not work in *Aspergillus* even at concentrations much higher than those effective in *S. pombe*. We can think of alternative strategies, but they fall beyond the time frame of a revised version of this manuscript.

The text makes the statement that fimA loss of function disturbs actin at the SPK even though fimbrin does not localize there. Does the SPK still exist in fimA::Tn341?

Please see my comment on the inappropriateness of using FM4-64 to reproducibly visualize the SPK in *Aspergillus nidulans*. We have used the myosin-5 instead, in this and several other mutants. With the help of this additional marker we have detected a weak signal of actin in approximately 50% of the tips (Figure 7E and F). However, the SPK is markedly affected, as in essentially all the tips MyoE localizes to a crescent, which contrasts with its localization in the wild type to an apical spec (see figure 1F). In addition, we have constructed a strain carrying the fimbrin mutation and expressing synaptobrevin-GFP (synaptobrevin is a component of secretory vesicles that accumulate in the SPK) (supplemental Figure 5). The SPK is not detectable in the mutant using this reporter.

My understanding is that the SPK is a structure that forms for fast hyphal growth. The slow growth of the fimA disruptant may indirectly perturb the formation of the SPK.

Yes, the referee is right, slow growth reflects less efficient endocytic recycling meaning that the SPK is debilitated. We have introduced this on p16.

**Minor comments:**

Are C-terminally tagged tropomyosins functional, or do they require co-expression of untagged tropomyosin. It would be helpful to make this clearer.

The wording of this particular section of the manuscript indeed required clarification. We have rephrased the sentences on page 6 and introduced a supplemental figure (S1 FIG) showing that tropomyosin–GFP expressed after endogenous tagging of the gene is unable to sustain viability.

is not very clear what the semi-structural illustration in Fig 1A shows. It looks like it has a white box covering part of the tropomyosin structure.
This has been corrected, many thanks.

Figure 2E is not very clear. If this is to illustrate that the endocytic collar stays at constant length from the apex over time, it would be helpful to use a rainbow color scheme to label time frames instead of black-and-white projection.
Thank you for calling our attention to this shortcoming. This panel, currently figure 1K in the revised version due to our rearrangement of items in Figures 1 and 2, has been substituted by a montage of the individual frames.

It is not clear to this reviewer how the LatB treatment affects microtubules. In Fig 1G, there is one MT ending at the SPK, but other MT seem to end elsewhere. What is the ground to state that MTs ends are disorganized upon actin depolymerization? If the authors want to make this statement, it should be backed up by some quantification.

Thank you for this comment. The problem of this section was that it had been extensively condensed and that the images supporting our statements were not particularly informative. Therefore, in addition to rephrasing these parts of the manuscript, we have introduced much better images which show that the dispersion of the MT ends across the swollen hyphal tips treated with latB is quite noticeable. Please see new figures 2B, D and E as well as new supplemental figure S2 and the corresponding text in the results.

In slaB conditional depletion, it would be more informative to show a kymograph of actin than a static image (5F), as this just looks like actin patches but does not show what the text states (clumps hanging from the cortex representing unproductive events...).
Thank you for noticing this shortcoming. We have taken two actions: we have introduced a movie showing what the statement says (supplemental movie 14) and a figure panel (6G) with a montage showing the lack of progression of actin patches in this mutant condition.

Line 389: “to” should read “two” changed, thank you

Line 460: The hypothetical statement that hyphal tip growth might resemble protrusion of filopodia is slightly odd. Tip growth is very well understood in fungi, and, besides involvement of formins and barbed end orientation towards the plasma membrane, does not resemble filopodial growth. I would encourage authors to remove this sentence.

We have removed that sentence. Thank you.

Is the naming of verprolin as vpr1 (rather than vrp1 in yeast) a typo? This is throughout in the text, but variable (vpr1 or vrp1) in figures.
We have corrected this typo throughout the MS, many thanks indeed

Figure S2C is not described in the legends.

Corrected in current figure S7 Fig

************************************************************************************

Reviewer #2: The actin module of endocytic internalization in Aspergillus nidulans: a critical role of WISH/DIP/SPIN90 family protein Dip1

The paper is a thorough and comprehensive fungal cell biology study that is interesting and important. The conclusions are predominantly well supported by experimental evidence but there are some areas which could be improved. These are summarised below.

• The authors refer to some of their findings as novel when in some instances they have been long known and, indeed, already published.

We apologize for this. We have made our best in this revised version to make it very explicit when we are referring to results that are confirmatory of data previously published by other laboratories.

• If the Chromobody gives “more accurate” numbers for actin patch lifetime, why did the authors choose to use Lifeact-FP instead in so many experiments? What are the advantages/disadvantages of the 5 actin reporters used?

I am afraid I cannot find a place where we said that chromobody gives more accurate numbers for actin patch lifetimes. The only distantly related statement was that the mCherry labeled version of chromobody gave a slightly shorter actin patch lifetime, and the actual statement referred to our interpretation that this occurs because this reporter is particularly sensitive to photobleaching.

• The authors make claims about the SPK but do not show it. I suggest carrying out FM4-64 staining in the strains labelled with Lifeact-FP in the mutant backgrounds. Or utilise a SPK reporter such as MyoA or SepA.

Thank you for the comment, which prompted us to perform new experiments that yielded interesting data. As noted in the cover letter for the editor, we have not used FM4-64, despite the fact that I pioneered its use in *A. nidulans* (Penalva 2005 PMID: 16291501). In *A. nidulans* FM4-64 labels mitochondria very strongly (Fischer-Parton, 2000, PMID: 10849201), making it difficult to distinguish the SPK as mitochondria very often colonize the tip region. Eventually one comes across a good picture, but these are exceptional. One of them is displayed in current figure 1E. In our experience, FM4-64 is not appropriate to get an overview of a hyphal population, and therefore we have followed the suggestion of using MyoE instead. We have now introduced experiments with MyoE and Lifeact GFP onto Figure 1F, Figure 7, Figure 9, Figure 13, Figure 16B and with SV cargoes on Figure S5 and Figure S8E, which with their associated text cover all instances in which we were kindly requested to use an independent marker for the SPK. Only one case we have used FM 4-64, which is the case of cofilin-GFP, that we photographed in a diploid, and therefore this is strain is not immediately amenable to genetic manipulation. This experiment is displayed on Figure 8D

• The WT vs dip1Δ model they propose at the end of the paper can be improved. The cartoon is very simplistic, poorly made with spatial/proportion and important inaccuracies. This should be improved.

Following experiments requested by referee one, we have concluded that both worms and actin cables emanating from the SPK are dependent on the activity of formin. Because actin patches still persist after inactivating formin, and even after inactivating formin in a *dip1∆* background, the alternative mechanism of providing seed filaments is currently unclear. Therefore, we have chosen to remove the model, as it is excessively speculative to be displayed as a conclusion. Thank you very much.

Line 135- The should add the [22] reference for N. crassa. added
Line 136- why is it problematic to observe the “exocytic” cables with Lifeact in A. nidulans in contrast to M. oryzae (Li et al., 2020, Plos Pathogens)?

Thank you. This was indeed poorly phrased. We substituted the word problematic by elusive and discussed that Lifeact efficiently detects cables in Magnaporthe.

Line 179- For clarity, specify what combination of sucrose+glucose is used in Figure 1C, either on the figure itself or the figure’s legend. Added in the figure legend of figure 1C.

Line 189- It is worth mentioning that 1.3 μm is the distance of the collar from the apex in A. nidulans, but that it is different in other fungi (e.g. N. crassa). We clarified that this figure refers to *A. nidulans.*

Line 202- How long did the hyphae take to recover after the drug wash out in each case?

The first frame of this movie was acquired 6 min after washing out the drug. This panel has been moved to S2 figure two have more space for display items. The time is now in the figure legend.

Line 229- For consistency, I suggest sticking either to collar or ring. Personally, I think collar is widely used in the past, and that way confusion is avoid (CARs).

This is a fair point; we have changed ring by collar everywhere

Line 236- The presence of F-actin in the core of the SPK has been known since many years, what is the novelty? In the text is understood as if it was sort of a new finding.

Indeed. This was very poorly worded. We have introduced a sentence to indicate the above fact.

Line 291- It seems the authors use the words ‘filament’, ‘cable’, ‘mini filament’, and ‘micro filament’, interchangeably, which is incorrect. Cables, like the other actin higher order structures (patches, SATs, CARs) are composed of F-actin. Therefore, the “worms” are not chunks of filaments, but rather chunks of cables. Please correct throughout the text the use of these words.

We have corrected this everywhere, excepting a couple of cases where we actually meant microfilaments

Line 348- Endocytosis in the subapical collar is unlikely to be clathrin-mediated (Schultzhaus et al., 2017, Mol. Microbiol.; Martzoukou et al., 2017, eLife). Do the authors have any explanation to this? Especially, since they mention that the uncoupling of the clathrin module and the F-actin module is the reason why the mutation of slab is lethal. This should be discussed.

We respectfully disagree with the referee’s statement that ‘endocytosis in the subapical collar is unlikely to be clathrin-mediated’, mentioning two *Aspergillus* papers to support this point.

(i) The physiological involvement of clathrin in endocytic internalisation in the budding yeast has been a subject of discussion for several years. Early reports documented that there is still endocytosis in the absence of clathrin *(Payne, G.S., Baker, D., van Tuinen, E., and Schekman, R. (1988). Protein transport to the vacuole and receptor-mediated endocytosis by clathrin heavy chain-deficient yeast*. *J Cell Biol 106, 1453-1461).*  The issue has been recently resolved by *Kaksonen et al., (Kukulski, W., Picco, A., Specht, T., Briggs, J.A.G., and Kaksonen, M. (2016). Clathrin modulates vesicle scission, but not invagination shape, in yeast endocytosis. eLife 5, e16036 and references therein)*, who explained the non-essential role of clathrin in the budding yeast. Therefore, the fact that clathrin is not essential for internalization does not necessarily imply that clathrin does not play any role in endocytosis.

(ii) In their seminal 2005 paper in Cell, Kaksonen et al. (2005) reported the difficulties that imaging FP-tagged clathrin in endocytic patches involved, due to strong internal signal of clathrin at the TGN, which masked cortical signals associated with endocytic patches. These problems were solved by using TIRF rather than conventional epifluorescence. Schultzhaus et al. (2017), mentioned by the referee, experienced similar problems with *A. nidulans*, yet they documented (with conventional epifluorescence) the presence of clathrin in the subapical collar (Figure 4A and several movies in this reference).

(iii) In the second paper mentioned by the referee, Martzoukou et al. , 2017 studied the physiological roles of AP-2, which in higher eukaryotes is a key endocytic adaptor involved in the formation of clathrin-coated endocytic vesicles. Martzokou et al , 2017, provide solid evidence that the physiological role of *A. nidulans* AP-2 (which is essential for polarity maintenance) is clathrin-independent, yet there are other proteins that can serve as adaptors between the endocytic coat and clathrin, and indeed these authors also studied the endocytic role of clathrin to conclude in the same paper that (literally) ‘An essential role of clathrin in the endocytosis of transporters is also well supported by our results’.

Therefore, while we appreciate that the referee was calling our attention to a potentially misguiding sentence, we believe that the debate on the relative importance of clathrin-mediated vs. clathrin-independent endocytosis in Aspergillus is premature, and requires a deeper understanding of the components and order of events governing this process. In this context, the role of clathrin in endocytosis in *Aspergillus* is a relatively ambitious objective that we will possibly pursue in the future, resources permitting.

Line 359- The absence of fimbrin in the SPK has been known for a very long time and yet istreated as a notable “novelty” in the text when it is already well established.

We have reworded this paragraph to confirm the somewhat confirmatory nature of these experiments, although we note that in the original report describing the localization of fimA -GFP in *Aspergillus nidulans* (ref. 4) the authors used an overexpression construct, in the presence of an untagged version of FimA. Specifically, in the case of the SPK, this paper does not mention explicitly that fimbrin is not detected at its position, yet we have also used the word confirmed to satisfy the referee. The referee should note that in this same report, the authors did not detect actin in the SPK.

Line 370- Is there still a SPK? Please add an image of the mutant with FM4-64 (or labelled with a SPK reporter) to show if this is an event of loss of polarity, what happens to the SPK? Maybe the Lifeact is still associated with the SPK, and this just has a different morphology in the mutant.
Thank you; this comment has been addressed above. There were indeed changes in the morphology of the Spitzenkörper imaged with MyoE.

Line 389- Typo “to” or make sentence clear

corrected.
Line 396- “Cofilin is essential” for? Section title could be more descriptive.

… viability. Thank you for catching this.

Line 588- Correct to mChFP, instead of mCh. Also correct throughout the text.

replaced
Line 600- Do you mean pathways or proteins?

Thank you for noticing the incorrect grammar

Line 638- Exocytosis should also be mentioned, as it is essential for hyphal tip growth.
Done

Line 646- While endocytosis studies are still scarce in filamentous fungi, I think insufficiently is not the correct word. There have been a number of good efforts to understand this process in the last decade and a half.

Modified

Line 672- Are they really yeast-like? It’s just a sick abnormal germling and doesn’t look like yeast to me.

Clarified
Line 685- The association of patches and cables has been already observed before in N. crassa (Delgado-Alvarez et al., 2010, FGB) and T. atroviride (Garduno-Rosales et al., 2022, FGB), which should be added in the discussion.

Thank you very much for catching this. These two references have been added in the context of this description.

Line 686- In the text, authors only called them “worms”, for consistency modify to “actin worms” in all the text.

changed
Line 700- Is it really striking? Why? Please justify.

It isn’t. Deleted.

Line 708- This needs further evidence to be stated that way. Justify.

We have removed this model for the resubmission. As noted above, we are not happy with the highly speculative model.

Line 722- “Filaments/cables” incorrect.

Corrected, thank you

Line 770- tdTomato.

Corrected, thank you
Line 784- Add space between the dot (.) and Reacting.

Corrected, thank you.

Line 795- Correct micrograms and micromolar to their short version.

Corrected, thank you.

Line 824- Add the camera that was used to capture the images.

Added

Line 902- Please correct the space between Lifeact and the hyphen.

Corrected

Line 920- More explanation is needed; the kymograph is not very clear for readers here.
We have added the necessary information. We have also introduced calibration bars for time and distance, which were inadvertently missing. Many thanks indeed

Line 936- Doesn’t look like yeast.

Swollen tip typical of a defect in polarity maintenance.

Line 943- For some colourblind individuals is actually very hard to distinguish between and cyan. Choose a colour combination that avoids confusion.

Cyan changed to yellow

Line 975- Consistency “actin collar”.

Changed
Line 984- Show FM4-64 image.

Under the heading above on general comments to the comments of the we argue why using FM 4-64 is far below being optimized. Throughout this revision we have used MyoE instead. On revised figure 9 panel B we show that the SPK is completely disorganized, as visualized with MyoE, which forms a crescent rather than localizing to an apical spec. Under these conditions an apical signal of F-actin was undetectable.

Line 1066- Correct “acting”., 1073- Correcy capital letter on “In”., line 1377- Where is C?
Thank you very much indeed. Corrected.

Figure 1- Using green/red palette should be avoid to support colourblind individuals. Please correct in all the pertinent figures. Does Lifect not label septation events in A. nidulans? Same for the other fluorophores. Why only showing it with Chromobody?

Green red palette substituted by green magenta palette throughout the manuscript Images of the contractile actin rings have been added to all figures Colors have been modified according to the suggestion of the referee. Images of CARs have now been added for all reporters, and panels from former figure 2 have been moved to this figure for convenience. Amongst these there is a new composition showing that the SPK labeled with Lifeact-GFP colocalizes with MyoE, demonstrating that the Lifeact signal indeed corresponds to this structure (Fig. 1F).

Figure 2- Please state what MIPs are for the readers as it is the first time using that abbreviation in the paper. Stated

For (F) would it be possible to colour differently the SPK from the patches to distinguish each population on the front image?

This image was difficult to interpret. We are now showing individual frames of a time-lapse sequence, but note that these have now been added to figure 1.

For (G) the figure itself is written as y,z, but in the figure legend is written as z,y. Please arrange for consistency. Corrected.

Also, there is no measurement of co-localisation. Calculate Pearson’s or Manders’ coefficient to quantify real co-localisation.

We respectfully believe that quantifying colocalization based on, for example, the Pearson coefficient, does not make sense, particularly when the informative pixels are very few. In view of the several comments raised by the referees, we have expanded this figure notably, with microtubules and actin now being considered in revised figure 2 and supplemental figure 2. Figure 2B shows one microtubule arriving at the SPK, but others arrive nearby, yet outside the SPK (S2 Fig).

In (I) is the collar really not present? Or is it just “moved to the tip”? A z-stack would probably give better evidence.

We provide the requested z-stack. The collar is dispersed and actin patches move towards the tip, both things happen.

In (H-I) what concentrations of the drugs were used? Indicate in figure and/or legend.

Added
In (J) the numbers (time stamps) are in reverse, correct.

Corrected, thanks.

Figure 4- In panel (E) where is the tip? Indicated.
(G) is not self-explanatory. Explanation provided in the legend

Figure 5- In (A) what is the point of showing the two FP? Pattern is the same.

Figure 6 in the revision. One has been deleted.

Figure 6- If the dotted line is accurate and it indicated the hyphal profile, then the hypha could be stressed (swollen tip), showing an endocytic collar further away from the tip than usual. Change image. The line was inaccurate it has been corrected.

On (E) indicate the mutant background in the image.

Indicated.

Figure 7- This should be a supplemental figure.

We have just removed it, showing quantitation in the respective mutant individual figures, as suggested by referee three.

Figure 8- On (B) the blue letters get lost with the background, please use a more contrasting colour. Corrected, thank you
On (D) What happens to the SPK? Using FM4-64 would help to resolve this. Because this is a diploid strain we did not introduce MyoE by crossing to label the SPK. In a few examples, one of which is shown, we detected the FM 4-64-labeled SPK colocalizing with a spec of F-actin

Figure 10- In (E) the right panel looks as if it was an offset between the two proteins. Might be better or separate the images and then show the merge. We followed the referee suggestion

Figure 13- Images in panel (C) should be stained with FM4-64 to show the SPK. We used MyoE instead (revised figure 13 panel E)

Figure 14- In (C) please correct for consistency to mChFP. Corrected

Figure 15- In (J) please indicate where the tip is. Done. Now supplemental figure 9F.

Figure 16- The endocytic patches look almost outside the hyphae. Please improve this figure. We have deleted the model from the revised version, we believe that with the new experiments it becomes excessively speculative, see general comments above.

Supplemental figure 3- In (A) why is the WT 37o so irregular in comparison to the one in supplemental figure 4? The figure 4 growth test mentioned by the referee was made on complete medium to maximize the chances of *sepA1* to grow at 37°, whereas that in former supplemental figure 3 was made in minimal medium, and possibly because the stabbing of the toothpick used to replicate the colony was not completely vertical. It does not affect the conclusion. The media used in each particular case is now in mentioned in the legends to figures.

Supplemental figure 5- Are those fungi grown at different temperatures? Indicate.

Thank you for catching this. Indicated.

****************************************************************

Reviewer #3: The manuscript entitled ‘The actin module of endocytic internalization in Aspergillus nidulans: a critical role of the WISH/DIP/SPIN90 family protein Dip1’ by Delgado & Pinar and collaborators is an astonishing piece of work in which a large number of proteins involved in the endocytosis of the model filamentous fungus Aspergillus nidulans are characterized. The authors use three criteria: 1) residence of F-actin in endocytic patches, 2) growth, and 3) endocytic recycling efficiency; to increase knowledge about the endocytic process in fungi showing that endocytosis is essential and providing relevant information on how F-actin drives the internalization of endocytic vesicles. Importantly, the work demonstrates a key role of Dip1 in supplying seed filaments for the formation of F-actin branching, although it makes it clear that is not the only one. The experiments are well designed and superbly executed and interpreted. The conclusions are clear and solidly supported. Among all the results, the in vivo fluorescence microscopy studies are simply incredible. For all these reasons I strongly recommend its publication.
Thank you very much for your kind words.

I include a series of non-essential suggestions that I believe would contribute to improving the final version of the manuscript:

1) Summary: In my opinion it is too specialized. I think it would be better to format it to make it easier for the general public to understand. Furthermore, it contains abbreviations such as FPs that are not indicated.

We have rephrased the abstract to make it more accessible to the general public and to incorporate the new findings resulting from experiments carried out in the revision period.
2) Colocalization studies in kymographs: The authors perform different colocalization studies using Lifeact (SlaB, ArpC1) or ArpC1 (Vrp1) but others are missing. I believe it would be very interesting to see when the explosion of F-actin occurs in the cycle of other proteins, particularly those that have a very different period.

We had included data on SlaB in the manuscript to compare the burst of F-actin with the lifecycle of a coat component. To satisfy the kind request of the referee, we have now included data on another coat component, End3 (text and supplemental figure 4R).

3) SlaB under the control of nitrate-inducible, ammonium-repressible promoter: I think it would be fantastic and informative to film, if possible, how growth and coupling of the clathrin and F-actin polymerization modules are restored by exchanging ammonium for nitrate…,

We have successfully filmed how germlings of the strain carrying the conditional expression allele of SlaB transit from the polarity maintenance defect to normal hyphae upon shifting them to nitrate, nicely illustrating how this recovery correlates with the reorganization of the actin cytoskeleton, notably of the endocytic collar. This is now shown on Figure 6H and supplemental movie 15. Thank you very much indeed for the suggestion.

Minor points: all addressed
1) Line 85: change ‘. in’ to ‘. In’.
2) Line 87: change ‘[4-6] Endocytic’ to ‘[4-6]. Endocytic’.
3) Line 172: change ‘[47] [48]’ to ‘[47, 48].
4) Line 303: change ‘minfilaments’ to ‘minifilaments’.
5) Line 312: change ‘that If these’ to ‘that if these’.
6) Line 388: change ‘to’ to ‘two’.
7) Line 479: change ‘in a narrow a spot’ to ‘in a narrow spot’.
8) Line 486: change ‘(Figure 11B).’ to ‘(Figure 11B),’.
9) Line 920: change ‘supplemental movie 11’ to ‘supplemental movie 9’.
10) Fig 4G: Point out the microfilament moving towards the tip for clarity.

11) Fig 7: I would move this figure to supplementary. I find it odd to see data for mutants that haven’t yet been discussed in the text. Another alternative (maybe better) would be to split it up to include the data for each mutant at the point where it’s discussed (always including the wt for clarity).

We have followed the first suggestion. Thank you
12) Fig 9E: Indicate what the red arrow means.

The red arrow marks the successive positions of the trailing end of this actin worm. Incorporated into the legend of the figure
13) Supp Fig 2: Revise the legend, (c) is missing.

Revised in the current supplementary equivalent S7 Fig)
14) Supp Fig 5: Letters are missing in the figure. Corrected
15) Results section ‘Actin polymerization occurs on SlaBSla2 patches’: Indicate that AbpA and SlaB are tagged endogenously. Indicated, thank you.
